# Transcriptome profiling of the rice blast fungus during invasive plant infection and *in vitro *stresses

**DOI:** 10.1186/1471-2164-12-49

**Published:** 2011-01-19

**Authors:** Sandra M Mathioni, André Beló, Christopher J Rizzo, Ralph A Dean, Nicole M Donofrio

**Affiliations:** 1 Department of Plant and Soil Sciences, University of Delaware, Newark, DE, USA; 2 WuXi AppTec, Inc., Philadelphia, PA, USA; 3 Department of Plant Pathology, Center for Integrated Fungal Research, North Carolina State University, Raleigh, NC, USA

## Abstract

**Background:**

Rice blast is the most threatening disease to cultivated rice. *Magnaporthe oryzae*, its causal agent, is likely to encounter environmental challenges during invasive growth in its host plants that require shifts in gene expression to establish a compatible interaction. Here, we tested the hypothesis that gene expression patterns during *in planta *invasive growth are similar to *in vitro *stress conditions, such as nutrient limitation, temperature up shift and oxidative stress, and determined which condition most closely mimicked that of *in planta *invasive growth. Gene expression data were collected from these *in vitro *experiments and compared to fungal gene expression during the invasive growth phase at 72 hours post-inoculation in compatible interactions on two grass hosts, rice and barley.

**Results:**

We identified 4,973 genes that were differentially expressed in at least one of the *in planta *and *in vitro *stress conditions when compared to fungal mycelia grown in complete medium, which was used as reference. From those genes, 1,909 showed similar expression patterns between at least one of the *in vitro *stresses and rice and/or barley. Hierarchical clustering of these 1,909 genes showed three major clusters in which *in planta *conditions closely grouped with the nutrient starvation conditions. Out of these 1,909 genes, 55 genes and 129 genes were induced and repressed in all treatments, respectively. Functional categorization of the 55 induced genes revealed that most were either related to carbon metabolism, membrane proteins, or were involved in oxidoreduction reactions. The 129 repressed genes showed putative roles in vesicle trafficking, signal transduction, nitrogen metabolism, or molecular transport.

**Conclusions:**

These findings suggest that *M. oryzae *is likely primarily coping with nutrient-limited environments at the invasive growth stage 72 hours post-inoculation, and not with oxidative or temperature stresses.

## Background

*Magnaporthe oryzae *is the causal agent of rice blast, the most threatening disease of cultivated rice worldwide. Spores of this filamentous ascomycete fungus, after landing on the leaf surface, form a germination tube. This tube senses the hydrophobicity and hardness of the host surface resulting in the formation of a penetration structure called an appressorium [1]. The accumulation of melanin in the appressorium cell wall and subsequent increase of glycerol levels in its interior generates high turgor pressure [2], which then leads to the formation of a penetration peg, a specialized hypha that is responsible for puncturing the plant epidermis and entering the plant cell [3,4]. Once inside the host, the fungus forms intracellular invasive hyphae (IH), from which filamentous hyphae emerge and follow a cell-to-cell growth pattern [5]. As a hemi-biotrophic organism, *M. oryzae *initially develops an intimate relationship with its host and in compatible interactions is presumably able to deal with any defenses the host may mount. Subsequently, host cells die resulting in characteristic "blast" disease symptoms.

Over the last decade, research in *M. oryzae *has largely focused on the morphological and physiological development of the fungus in the pre-penetration and penetration phases as reviewed by Howard and Valent [3]. Genome-wide gene expression analyses of *M. oryzae *have examined early-stage events, including spore germination and appressorium formation on a hydrophobic surface and in response to cyclic AMP induction [6]. Other studies have focused on a single element in isolation, such as response to nitrogen-starvation, or effects of single gene mutation [7-9]. Recent studies have examined the early invasive phase of the pathogen within the plant cells, revealing intriguing results on the truly hemi-biotrophic nature of this fungus [5,10,11]. A transcriptome analysis of *M. oryzae *at an early stage of invasive growth (36 hours post-inoculation, hpi) identified a number of so-called biotrophy-associated secreted proteins [10]. On the other hand, there is a paucity of information on the challenges the fungus potentially faces during invasive growth in the host environment. Furthermore, very little is known about global gene expression changes when the fungus is exposed to various environmental stresses. We postulate that *M. oryzae *must have evolved mechanisms for coping with various host conditions--some potentially stressful--as infection progresses. Some of the earliest plant responses upon pathogen attack are the production of reactive nitrogen species (RNS; [12]) and reactive oxygen species (ROS; [13]). High levels of these substances can be extremely toxic and can cause oxidative damage to DNA, RNA, lipids, and proteins if not catalyzed by the cell [14]. Evidence supporting the idea that plant pathogens encounter RNS and ROS comes, for example, from the barley obligate biotroph fungus, *Blumeria graminis *f.sp. *hordei*, from which the synthesis and secretion of antioxidant enzymes, such as catalases and peroxidases, was observed during germ tube invasion in a compatible interaction [15].

The search for nutrients is also a challenge faced by pathogens during host infection [16,17]. During *in planta *growth and development of filamentous intracellular hyphae, a close relationship with host cells is formed, enabling the uptake of nutrients, such as carbon and nitrogen by the pathogen, as well as secretion of proteins into host cells [18]. In *M. oryzae*, nitrogen availability appears to regulate pathogenicity. NPR1 and NPR2, regulators of nitrogen utilization [19] also regulate *MPG1*, which encodes a small hydrophobic protein. *MPG1*, which is required for pathogenicity, is induced under carbon and nitrogen limiting conditions and during *in planta *growth. These data suggest that *M. oryzae *encounters nutrient limitation during infection. Other evidence for the nutrient challenge faced by plant pathogens was shown by Voegele and colleagues [20]. They identified a hexose transporter (*HXT1*) that is localized in the haustorial plasma membrane and highly expressed in the haustoria of the rust fungus *Uromyces fabae*, a common pathogen of beans. The HXT1 has high affinity for D-glucose and D-fructose substrates, suggesting that these sugars are taken up by the haustorium during the fungus-plant interaction.

One strategy to explore the likely environmental challenges faced by the fungus during later stages of host invasive growth is to analyze the global transcriptome of the pathogen during growth *in planta *compared to growth during *in vitro *stresses. We hypothesize that the function of genes, which have common expression patterns during *in planta *infection and *in vitro *stresses, will likely represent the molecular mechanisms the fungus utilizes for successfully coping with its host environment. We used microarrays to examine the expression of approximately 11,000 *M. oryzae *genes during *in planta *compatible growth (rice and barley at 72 hpi) and *in vitro *stresses (temperature up shift, oxidative stress, and nutrient limitation) compared to fungal mycelia grown in complete medium (reference sample). We generated a robust and diverse dataset that had not yet existed for *M. oryzae*, which we then analyzed to identify genes commonly induced or repressed in combinations of treatments. The data are further discussed in the context of gene functionality, their role in fungal physiology during *in planta *invasive growth and the utility of this dataset for hypothesis-building.

## Results

### Microarray data analyses and validation

Our primary interest in undertaking this gene expression profiling experiment was to ascertain whether gene expression patterns during *in planta *invasive growth were similar to *in vitro *stress conditions, and which condition most closely mimicked that of *in planta *invasive growth. To this end, we performed microarray experiments with RNA samples collected from eight conditions, two from *in planta *invasive growth in two-week old rice (R) and barley (B) leaves at 72 hpi (hours post-inoculation), and six from *M. oryzae *axenic cultures grown in complete medium and then subjected to temperature up shift at 42 °C (TS) for 45 minutes, oxidative stress using 5 mM of the oxidative agent Paraquat (PQ) for 24 hours, minimal medium (MM), carbon-limited minimal medium (MM-C), and nitrogen-limited minimal medium (MM-N). We chose to examine 72 hpi because this is the most likely stage at which invasive growth is occurring; that is, the fungus spreads to adjacent epidermal cells and the first visible signs of disease begin to appear [21]. We based our choices for the *in vitro *experiments on a combination of preliminary data and previously published studies. For example, the time-point and temperature for TS was chosen based upon a published study on chaperone-related gene changes in *M. oryzae *subjected to a heat shock of 42 °C longer than 40 minutes, [22] as well as prior experiments that looked at induction of Hsp104 (Donofrio and Dean, unpublished results; refer to Methods for further details on conditions chosen).

*M. oryzae *grown in complete medium was used as the reference condition for each hybridization experiment (see Methods for details and rationale). The microarray data were analyzed using the Limma software package ([23-28]; see Methods for details). The probes were considered to represent significantly differentially expressed transcripts if they had a minimum of two-fold expression, either up or down, in relation to the reference in at least one of the treatments (P-values < 0.01), and presence of a MGG number in version six of the *M. oryzae *genome annotation database [29]. Using these criteria, we identified 4,973 genes differentially expressed among the conditions tested when compared to the reference sample (Table 1). The rice, barley and MM-C treatments had more induced than repressed genes while the opposite was observed for the other *in vitro *treatments (TS, PQ, MM, and MM-N). Similarly, rice, barley, MM-C, and MM-N had more unique genes being differentially expressed than TS, PQ and MM (Table 1; the details for the ten most induced and repressed genes for each treatment are available in Additional file 1).

**Table 1:** Number of differentially expressed genes in each stress condition

	R* (72 hpi)	B (72 hpi)	TS	PQ	MM	MM-C	MM-N
FC > 2	1121 (10.1)	1784 (16.0)	720 (6.4)	694 (6.2)	485 (4.3)	1111 (10.0)	1012 (9.1)
FC < -2	760 (6.8)	1223 (11.0)	998 (8.9)	887 (7.9)	731 (6.5)	593 (5.3)	1119 (10.0)
Unique (FC > 2)^A^	234 (2.1)	449 (4.0)	90 (0.81)	86 (0.77)	6 (0.05)	228 (2.0)	233 (2.1)
Unique (FC < -2)	117 (1.0)	246 (2.2)	82 (0.73)	105 (0.94)	4 (0.03)	91 (0.82)	222 (2.0)

*R (rice; hpi - hours post-inoculation), B (barley), TS (temperature up shift), PQ (Paraquat), MM (minimal medium), MM-C (minimal medium without carbon), and MM-N (minimal medium without nitrogen). FC (fold-change). In parenthesis is showed the percentage from the total number of genes in the genome (11,089 genes).

^A ^Genes differentially expressed only in that specific treatment.

In order to validate the microarray results, we selected 21 genes that were either induced or repressed in all treatments and measured the difference in expression of these genes in relation to *M. oryzae *grown in complete medium (reference sample) with quantitative reverse-transcription polymerase chain reaction (qRT-PCR; see Methods for details). The averaged matching between the microarray and qRT-PCR data was approximately 73% (Additional file 2). Four of the 21 genes did not match the microarray results and, in fact, provided exactly opposite results with high variability in the expression levels. We suspected that either the primers used in the qRT-PCR amplified other cDNAs or that the microarray probes cross-hybridized to other unexpected transcripts, although the former possibility is unlikely, given that we blasted all of the primer sequences to the *M. oryzae *database, and they only returned one correct hit to the gene of interest. After excluding those four genes, the overall percentage of matches between the two technologies was approximately 90% (Additional file 2).

**Figure 1:**
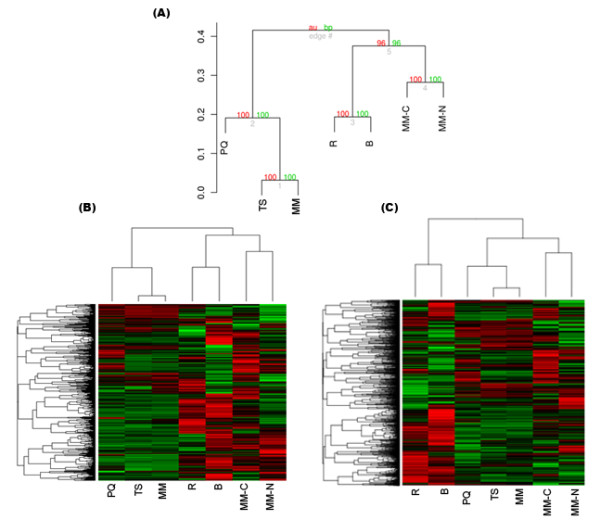
**Hierarchical clustering of *M. oryzae *treatments**. (A) Hierarchical clustering of gene expression in each of seven *M. oryzae *treatments based on the transcription profile of the 1,909 genes differentially expressed. The distance method used to cluster the treatments was 1 - correlation (*y*-axis). The red and green values in each node of the dendrogram represent the percentage confidence of the cluster estimated by 10,000 bootstrap re-samplings. (B) Heatmap of the set of 1,909 genes from panel (A), where red and green represent induced and repressed gene expression versus the reference sample, respectively. (C) Heatmap of the "meta-dataset" of 4,793 genes (red and green colors as above). Acronyms stand for fungal gene expression during the following conditions: R = rice at 72 hpi; B = barley at 72 hpi; PQ = paraquat (oxidative); TS = temperature up-shift (heat shock); MM = minimal media; MM-C = carbon limitation; MM-N = nitrogen limitation.

In order to identify fungal gene expression patterns from the *in vitro *treatments that were most similar to gene expression *in planta*, we further sorted the 4,973 differentially expressed gene set. Genes were grouped together if they had significant expression in rice and/or barley and at least one other *in vitro *condition, which resulted in 1,909 genes. Hierarchical clustering of this subset of genes revealed three major clusters (Figure 1A). The first grouped the *in vitro *treatments PQ, TS and MM, the second grouped the *in planta *treatments rice and barley, and the third grouped the nutrient-limited treatments MM-C and MM-N. Overall, we found that fungal gene expression in rice and barley more closely grouped with MM-C and MM-N, than the other *in vitro *stress conditions. Heat maps are shown for the smaller 1,909-gene dataset, as well as the "meta-dataset" of 4,973 genes (Figures 1B and 1C, respectively). Clustering of the meta-dataset revealed that fungal gene expression *in planta *was more disparate from any of the *in vitro *conditions, but was still closer to the nutrient-limited conditions, as indicated by the length of the tree branch.

### *M. oryzae *genes differentially expressed in all treatments

**Figure 2:**
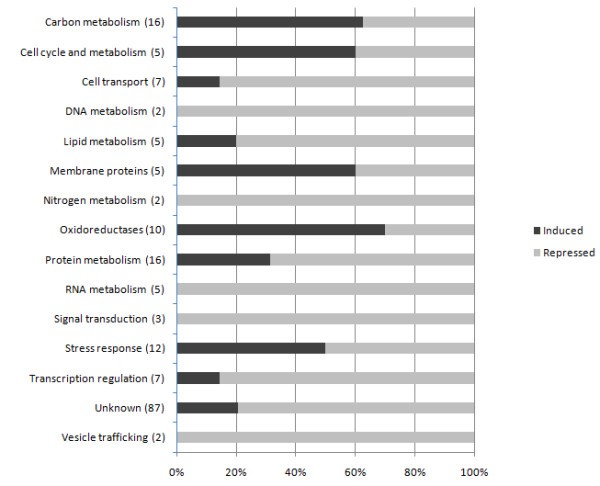
**Cumulative percentage of induced vs. repressed genes according to their gene ontology**. Functions of genes that were commonly induced (55) and commonly repressed (129) across all seven conditions were predicted using gene ontology (GO) and represent major gene functions in the fungal physiology and biochemistry. The total number of differentially expressed genes for each functional category is shown in parentheses.

We also wished to know whether any genes shared expression patterns among all seven conditions and the reference condition. From the 4,973 genes, 55 and 129 genes were commonly induced and repressed in all treatments (Tables 2 and 3, respectively). From the 55 induced and 129 repressed genes, 37 and 60 genes have known function, respectively. The genes with unknown function were subjected to BLAST searches at NCBI and the results are included in Tables 2 and 3. The 55 and 129 genes were grouped into categories based on their cellular function using Gene Ontology (GO). Under the stringent criterion of being induced in all seven conditions, we noted a higher percentage of induced genes involved in carbon metabolism, oxidation-reduction reactions and membrane metabolism than in any other category (Figure 2). Interestingly, there were no nitrogen metabolism-related genes that were induced in all seven conditions. Genes with putative roles in stress responses showed equal numbers being induced and repressed, as was also the case for genes involved in cell cycle. Among the induced genes in all conditions there were two glutathione S-transferases (MGG_05565.6 and MGG_06747.6), enzymes that play a role in cellular detoxification in yeast [30]; an endo 1,4-β xylanase (MGG_07868.6) and a cutinase (MGG_05798.6), enzymes involved in plant cell wall [31] and cutin [32] degradation in *M. oryzae*, respectively; and a homologue of the pisatin demethylase gene (*PD*, MGG_04404.6) which, in the pea (*Pisum sativum *L.) fungus *Nectria haematococca*, is involved in the detoxification of the phytoalexin pisatin [33]. Analyses of the pisatin amino acid sequence of *PD *and MGG_04404.6 revealed the presence of a ligand binding site that is present in the cytochrome P450 protein family, suggesting that MGG_04404.6 is another member of this protein family [33].

**Table 2:** List of the 55 genes induced in all treatments

		Fold change						
		
Gene	Putative function	**R**^**1**^	B	TS	PQ	MM	MM-C	MM-N
MGG_02239.6	Unknown	107.6	132.2	4.5	4.6	2.0	20.5	3.0
MGG_07868.6	Endo-1,4-beta-xylanase	86.8	387.5	4.4	4.6	2.6	39.6	4.2
MGG_04404.6	Pisatin demethylase	39.8	52.6	3.8	6.9	3.2	11.8	8.1
MGG_01941.6	FAD binding domain-containing protein	28.8	34.0	2.5	5.2	2.2	4.2	3.7
MGG_04522.6	Unknown	22.1	68.1	9.5	11.6	7.1	17.3	12.2
MGG_05798.6	Cutinase (1E-32)	20.2	53.1	3.3	3.0	2.3	8.1	5.4
MGG_08527.6	Nucleoside-diphosp-sugar epimerase (2E-86)	18.5	33.6	2.3	3.5	2.1	4.2	5.4
MGG_05954.6	Glycoside hydrolase family 79 protein (2E-21)	16.9	49.9	9.0	2.6	6.7	9.1	4.2
MGG_01863.6	Aminopeptidase Y	16.6	34.4	2.4	2.7	2.0	8.6	8.9
MGG_08583.6	Beta-glucosidase 1 precursor	15.9	27.4	2.5	2.3	2.2	3.2	2.6
MGG_02225.6	2,3-dihydro-2,3-dihydroxybenzoate dehydrogenase	15.3	22.7	5.2	5.5	4.5	4.9	4.3
MGG_05565.6	Glutathione S-transferase	14.2	29.9	5.5	4.8	3.5	7.9	5.5
MGG_14657.6	SAM-dependent methyltransferase (3E-29)	11.8	22.4	2.8	2.5	2.7	5.0	12.2
MGG_08985.6	Beta-xylosidase	9.3	58.5	4.6	3.9	2.4	17.7	9.3
MGG_09867.6	N-acetyltransferase ats1	8.2	9.5	4.7	4.6	4.5	3.4	2.6
MGG_05912.6	N-acyl-L-amino acid amidohydrolase	8.0	48.7	3.7	27.5	2.1	4.9	20.7
MGG_00039.6	Ketose-bisphosphate aldolase class-II (2E-117)	7.8	8.8	4.0	4.0	3.9	4.4	5.3
MGG_03900.6	Aldehyde dehydrogenase	7.7	23.0	3.2	4.1	3.4	9.1	3.7
MGG_09433.6	Endoglucanase family 5 glycoside hydrolase	7.7	38.7	3.3	4.8	2.9	7.6	3.2
MGG_02409.6	Non-specific lipid-transfer protein	7.3	30.2	2.2	2.2	2.1	8.2	2.2
MGG_06747.6	Glutathione S-transferase	6.9	8.0	3.6	3.6	3.1	17.2	2.4
MGG_02559.6	MOSC domain-containing protein	6.8	42.3	2.8	2.7	2.1	11.0	4.4
MGG_09757.6	Neutral alpha-glucosidase ab	6.4	17.8	2.6	3.9	2.5	6.8	5.7
MGG_01843.6	Phosphatidylethanolamine-binding protein	5.4	31.7	2.2	2.1	2.0	5.1	3.1
MGG_10518.6	(R)-benzylsuccinyl-CoA dehydrogenase	5.2	18.2	2.6	3.2	2.6	5.8	3.3
MGG_09602.6	Membrane copper amine oxidase	5.1	7.3	4.5	3.6	4.2	4.6	2.1
MGG_06586.6	Quinone oxidoreductase	5.1	4.5	2.2	2.7	2.2	2.9	6.3
MGG_13518.6	Sorbose reductase SOU1	5.0	6.4	3.1	3.3	2.6	7.1	9.6
MGG_07933.6	Dihydrodipicolinate synthase	4.9	10.6	2.8	8.4	2.7	6.3	12.3
MGG_04164.6	DUF427 domain-containing protein	4.7	28.1	7.3	7.0	6.4	12.3	5.3
MGG_09218.6	Dehydrogenase/reductase SDR family member 4	4.6	12.8	3.2	2.5	2.7	4.3	2.5
MGG_00663.6	Phytanoyl-CoA dioxygenase family protein	4.6	20.1	5.2	5.2	3.7	5.6	7.8
MGG_01920.6	C2H2 type zinc finger domain protein	4.6	18.6	4.0	5.7	3.5	6.4	5.9
MGG_01772.6	Thioesterase family protein (1E-23)	4.3	7.9	3.5	2.5	3.4	4.1	2.1
MGG_09352.6	Minor extracellular protease vpr	4.2	8.7	9.7	8.3	7.9	5.0	4.3
MGG_07629.6	Flavin-binding monooxyg-like protein (0.0)	4.0	6.7	2.7	3.6	2.6	4.4	2.9
MGG_14087.6	Glucan 1,3-beta-glucosidase	3.8	6.0	3.7	4.3	3.6	6.9	2.8
MGG_07627.6	Homoserine acetyltransferase family protein	3.7	10.0	3.3	3.9	3.2	12.0	15.3
MGG_08554.6	Amidohydrolase family protein	3.7	9.5	3.8	4.3	3.5	3.4	4.6
MGG_13765.6	Serine carboxypeptidase S28 protein (6E-163)	3.7	14.3	3.6	4.3	3.4	11.1	12.1
MGG_01081.6	Peroxin 14/17	3.5	9.1	3.6	2.6	3.2	3.9	2.0
MGG_06386.6	Unknown	3.1	33.5	2.2	2.4	2.2	11.5	5.9
MGG_06784.6	Aldo-keto reductase	3.0	19.4	3.3	2.0	3.2	6.7	6.6
MGG_03095.6	Dihydroxyacetone kinase	3.0	19.1	3.6	3.7	3.3	3.5	4.7
MGG_02330.6	Extracellular serine-rich protein (6E-23)	2.9	4.8	7.2	4.8	3.3	3.8	4.9
MGG_02169.6	Unknown	2.9	7.0	2.5	4.2	2.1	3.9	6.6
MGG_09979.6	Monooxygenase	2.8	7.4	3.2	3.0	3.0	4.2	2.4
MGG_03094.6	Triosephosphate isomerase 2	2.7	8.2	2.9	2.9	2.4	2.1	2.1
MGG_11530.6	MFS transporter (2E-89)	2.7	16.2	2.6	2.7	2.6	4.4	14.9
MGG_08306.6	Abhydrolase domain-containing protein 12	2.4	8.2	3.2	2.1	2.6	4.5	4.1
MGG_15269.6	DUF636 domain-containing protein	2.2	10.8	2.9	4.2	3.0	5.9	4.8
MGG_07761.6	Unknown	2.2	4.0	4.7	5.4	4.0	4.8	2.7
MGG_08611.6	FAD dependent oxidoreductase (4E-87)	2.1	4.5	2.3	3.0	2.3	3.2	2.6
MGG_07665.6	Unknown	2.1	5.5	2.9	2.3	2.8	13.2	11.9
MGG_01316.6	Unknown	2.1	14.8	8.3	4.3	6.5	7.0	13.3

^1^list sorted in descending fold-change in the rice treatment. R: rice at 72 hpi; B: barley at 72 hpi; TS: temperature up shift, PQ: Paraquat, MM: minimal medium, MM-C: carbon limitation, MM-N: nitrogen limitation. The adjusted P-values for these genes are < 0.01.

**Table 3:** List of the 129 genes repressed in all treatments

		**Fold change**^**1**^						
		
Gene	Putative function	R	B	TS	PQ	MM	MM-C	MM-N
MGG_07766.6	Unknown	-11.0	-63.6	-74.5	-99.2	-59.9	-31.0	-114.6
MGG_02709.6	Unknown	-7.5	-7.6	-20.1	-3.2	-15.6	-2.9	-6.8
MGG_00312.6	Glyoxylate reductase	-5.6	-8.9	-4.0	-3.1	-4.0	-2.4	-2.1
MGG_11617.6	Unknown	-5.6	-9.5	-42.2	-28.0	-52.5	-12.1	-20.0
MGG_03144.6	Ulp1 protease family protein	-5.4	-8.9	-11.6	-2.4	-10.3	-2.8	-3.9
MGG_09345.6	Aminotransferase (2E-94)	-5.4	-6.6	-9.7	-5.5	-7.6	-3.9	-7.2
MGG_09158.6	Unknown	-5.3	-17.8	-64.0	-60.0	-48.9	-11.0	-39.6
MGG_05994.6	Unknown	-5.3	-18.6	-4.4	-5.7	-4.6	-2.0	-4.6
MGG_00460.6	Rhamnolipids biosynthesis	-5.1	-6.3	-39.9	-24.5	-33.6	-6.5	-7.6
MGG_03416.6	Acetyltransferase	-4.9	-16.3	-2.4	-3.3	-2.5	-3.2	-3.4
MGG_07911.6	Methyltransferase	-4.6	-6.4	-36.1	-34.3	-25.5	-7.1	-16.3
MGG_11610.6	Unknown	-4.6	-6.5	-38.3	-24.7	-25.0	-8.4	-7.8
MGG_05985.6	Ankyrin/HET domain-containing prot (8E-44)	-4.5	-5.9	-10.0	-10.5	-9.7	-2.5	-5.4
MGG_03639.6	Arginyl-tRNA synthetase (5E-30)	-4.5	-8.1	-39.7	-27.5	-31.0	-10.5	-21.1
MGG_10704.6	GNAT family acetyltransferase (1E-25)	-4.4	-4.6	-7.5	-5.8	-6.8	-4.9	-14.0
MGG_09031.6	Transcriptional regulator (7E-65)	-4.3	-4.7	-28.0	-21.3	-18.1	-7.9	-14.5
MGG_05055.6	Alcohol dehydrogenase	-4.2	-6.0	-23.2	-9.9	-30.4	-6.2	-9.7
MGG_01668.6	Unknown	-4.2	-6.4	-5.0	-3.4	-4.8	-2.4	-3.8
MGG_01013.6	Eukaryotic translation initiation factor	-4.1	-4.6	-6.1	-6.3	-5.5	-2.3	-3.1
MGG_05001.6	RNase P and RNase MRP subunit (2E-10)	-4.1	-11.4	-7.5	-5.5	-7.3	-2.8	-6.4
MGG_10859.6	Linoleate diol synthase	-4.0	-5.9	-12.1	-10.5	-10.4	-4.7	-6.0
MGG_04048.6	Aspartic proteinase	-4.0	-4.8	-11.3	-11.0	-6.2	-2.9	-8.0
MGG_14917.6	Ring canal kelch protein	-4.0	-4.7	-2.7	-2.9	-2.5	-3.0	-3.0
MGG_08333.6	Unknown	-4.0	-5.9	-2.3	-2.5	-2.2	-4.2	-5.6
MGG_05914.6	Tyrosinase (1E-52)	-3.9	-7.0	-28.6	-15.5	-22.7	-9.5	-13.9
MGG_09255.6	Kinesin-II 85 kDa subunit	-3.8	-6.5	-9.4	-6.6	-7.2	-6.4	-4.8
MGG_00197.6	Unknown	-3.8	-7.3	-21.5	-7.4	-19.2	-2.1	-9.3
MGG_01825.6	Unknown	-3.8	-8.3	-10.6	-7.0	-10.4	-3.4	-4.9
MGG_01439.6	Inorganic phosphate transporter PHO84	-3.7	-3.5	-5.5	-5.0	-3.2	-3.8	-2.8
MGG_00791.6	Lactamase_B domain containing protein	-3.7	-5.7	-5.4	-4.6	-5.3	-3.6	-11.7
MGG_09326.6	Unknown	-3.7	-2.8	-7.6	-14.0	-5.7	-3.6	-5.6
MGG_03308.6	Unknown	-3.7	-7.2	-14.3	-7.0	-8.3	-4.3	-12.0
MGG_10755.6	Arylesterase/monooxygenase	-3.6	-10.7	-28.1	-23.3	-5.7	-5.9	-15.5
MGG_06470.6	DNA repair helicase RAD25	-3.5	-5.2	-7.7	-3.8	-7.4	-2.2	-3.2
MGG_09893.6	Molybdopterin synthase small subunit CnxG	-3.5	-6.9	-3.9	-10.7	-4.0	-9.1	-10.6
MGG_03651.6	Unknown	-3.5	-5.3	-18.3	-6.7	-17.1	-7.5	-9.0
MGG_04024.6	Putative b-zip transcription factor (4E-05)	-3.5	-9.1	-18.0	-14.3	-15.3	-4.5	-15.7
MGG_05100.6	PR-1-like protein (3E-18)	-3.5	-7.7	-34.4	-14.1	-15.2	-4.7	-13.0
MGG_00919.6	GMP synthase	-3.4	-5.3	-2.7	-2.3	-2.6	-2.9	-2.7
MGG_03690.6	Cholinephosphotransferase 1	-3.4	-3.3	-5.4	-6.7	-5.6	-3.7	-3.3
MGG_14045.6	Unknown	-3.4	-6.3	-3.9	-3.8	-3.7	-3.6	-10.3
MGG_08816.6	Unknown	-3.4	-8.4	-16.8	-13.8	-8.7	-5.9	-12.7
MGG_15446.6	Unknown	-3.4	-3.5	-4.2	-13.3	-2.4	-2.9	-4.7
MGG_00296.6	Glycosyl hydrolase	-3.3	-3.8	-9.4	-3.5	-7.8	-2.3	-5.9
MGG_12911.6	tRNA 2'-phosphotransferase 1	-3.3	-5.8	-6.1	-5.3	-5.6	-3.5	-6.8
MGG_04401.6	F-box protein	-3.3	-6.3	-10.7	-11.7	-10.5	-7.7	-11.7
MGG_05239.6	DNA repair and recombination protein RAD26	-3.3	-7.0	-5.7	-4.1	-5.4	-5.4	-4.6
MGG_02611.6	L-aminoadipate-semialdehyde dehydrogenase	-3.3	-4.3	-3.3	-2.5	-3.7	-3.2	-2.9
MGG_07950.6	Related to glyoxal oxidase precursor (4E-10)	-3.3	-3.2	-7.0	-8.8	-2.3	-3.8	-8.4
MGG_03415.6	Ankyrin repeat/SAM domain protein 6 (4E-58)	-3.2	-6.0	-3.2	-3.6	-3.1	-2.2	-2.6
MGG_04395.6	F-box domain-containing protein	-3.1	-3.7	-2.5	-3.6	-2.5	-3.8	-3.2
MGG_09221.6	Related to glyoxal oxidase precursor (3E-09)	-3.1	-14.6	-23.3	-18.4	-17.2	-5.3	-15.9
MGG_02762.6	ATP-dependent RNA helicase DED1	-3.0	-9.2	-7.5	-8.2	-4.9	-5.8	-12.0
MGG_01260.6	Serine/threonine-protein kinase psk1	-3.0	-4.3	-5.6	-10.2	-5.7	-8.0	-9.9
MGG_09990.6	Minor extracellular protease vpr	-3.0	-4.6	-19.2	-21.6	-16.6	-6.9	-11.3
MGG_09010.6	Transcriptional activator hac1	-3.0	-2.2	-2.8	-5.5	-2.5	-3.1	-3.3
MGG_03061.6	Ankyrin repeat-containing protein	-3.0	-6.0	-14.3	-13.6	-12.0	-5.8	-11.2
MGG_02489.6	Branched-chain-amino-acid aminotransferase	-3.0	-5.0	-3.6	-3.7	-3.5	-4.2	-3.3
MGG_02101.6	Methyltransferase type 11 (6E-45)	-3.0	-6.4	-3.0	-5.7	-2.9	-2.6	-8.5
MGG_01297.6	Flavin-nucleotide-binding protein (3E-66)	-3.0	-3.4	-12.4	-5.4	-11.2	-9.9	-12.8
MGG_01806.6	Nicotinamide n-methyltransferase (2E-12)	-3.0	-5.7	-2.8	-4.7	-2.5	-2.8	-6.6
MGG_06382.6	Meiosis-specific ser/threo kinase mek1 (2E-34)	-3.0	-5.9	-5.4	-6.4	-3.7	-2.8	-3.9
MGG_03138.6	Unknown	-3.0	-2.1	-6.2	-6.0	-5.8	-4.5	-3.4
MGG_06045.6	YIPF1 (4E-106)	-2.9	-2.9	-5.0	-11.5	-4.8	-4.1	-2.3
MGG_15240.6	Unknown	-2.9	-6.6	-5.4	-7.5	-3.6	-3.4	-7.4
MGG_05990.6	mRNA-capping enzyme subunit beta (1E-60)	-2.9	-3.9	-3.4	-4.7	-3.3	-4.0	-2.7
MGG_08700.6	SET domain-containing protein 5 (2E-15)	-2.9	-3.1	-25.7	-17.3	-17.2	-4.9	-11.3
MGG_07283.6	Ulp1 protease family protein	-2.8	-5.5	-4.5	-2.4	-4.0	-2.3	-3.1
MGG_09019.6	Secretory phospholipase A2	-2.8	-6.5	-23.5	-15.4	-18.8	-12.8	-19.4
MGG_07912.6	Erythrocyte band 7 integral membrane protein	-2.8	-4.7	-4.6	-4.1	-4.3	-2.8	-4.9
MGG_07233.6	Potassium transporter, putative (4E-114)	-2.8	-5.0	-3.3	-3.7	-3.6	-7.0	-7.7
MGG_01341.6	Zinc metallopeptidase (3E-91)	-2.8	-3.4	-4.0	-4.2	-3.7	-3.9	-3.2
MGG_10259.6	Unknown	-2.8	-5.9	-5.5	-5.6	-2.3	-3.3	-6.2
MGG_05254.6	Histone-lysine N-methyltransferase	-2.7	-3.6	-5.5	-7.5	-5.3	-5.1	-4.6
MGG_00419.6	Major facilitator superfamily transporter	-2.7	-2.7	-30.5	-20.8	-11.4	-8.8	-14.0
MGG_01014.6	C-1-tetrahydrofolate synthase	-2.7	-3.3	-7.3	-6.4	-6.6	-3.2	-3.5
MGG_07192.6	Unknown	-2.7	-3.1	-9.0	-7.5	-7.8	-3.1	-2.9
MGG_07949.6	Choline dehydrogenase (2E-27)	-2.7	-2.7	-14.5	-11.3	-15.6	-6.2	-12.2
MGG_07328.6	Stress response RCI peptide, putative (3E-21)	-2.7	-4.5	-2.5	-2.8	-2.3	-2.7	-3.3
MGG_00013.6	Unknown	-2.7	-3.1	-13.5	-9.6	-2.8	-4.2	-11.5
MGG_04595.6	Unknown	-2.7	-3.3	-9.7	-2.3	-6.2	-2.4	-7.7
MGG_05181.6	Cell wall anchored protein (1E-17)	-2.7	-2.8	-10.1	-7.7	-4.1	-7.0	-8.7
MGG_09827.6	Sugar transporter family protein	-2.6	-6.6	-8.8	-11.6	-8.1	-2.9	-10.0
MGG_01309.6	Histidine biosynthesis trifunctional protein	-2.6	-3.2	-3.5	-3.1	-3.6	-2.7	-3.8
MGG_06888.6	Glutamine synthetase	-2.6	-2.2	-3.5	-4.2	-3.1	-3.1	-6.2
MGG_00481.6	Glutamate 5-kinase	-2.6	-3.4	-5.3	-6.1	-5.2	-5.6	-3.7
MGG_04330.6	Mitochondrial ribosomal protein subunit S4	-2.6	-3.7	-2.7	-2.3	-2.6	-2.6	-2.5
MGG_04356.6	ATP phosphoribosyltransferase	-2.5	-4.8	-2.5	-3.1	-2.5	-3.0	-3.7
MGG_04868.6	Purine-cytosine permease FCY22	-2.5	-3.8	-4.5	-4.1	-4.0	-2.5	-2.9
MGG_13245.6	Translation regulator GCD7	-2.5	-5.3	-3.6	-3.0	-3.5	-2.1	-4.0
MGG_09068.6	NADPH-dep 1-acyldihydroxyacetone phos reductase	-2.5	-4.7	-15.2	-13.6	-6.1	-2.9	-7.2
MGG_01751.6	F-box and WD domain-containing protein	-2.5	-2.3	-2.2	-2.3	-2.3	-2.2	-2.3
MGG_02937.6	Histone-lys N-methyltransfer. (Ash1) (6E-88)	-2.5	-3.6	-4.5	-5.6	-4.9	-6.4	-4.2
MGG_03916.6	Unknown	-2.5	-3.4	-8.9	-8.6	-8.8	-2.9	-6.8
MGG_03698.6	Unknown	-2.5	-3.7	-2.5	-2.5	-2.6	-2.9	-2.1
MGG_10544.6	Integral membrane protein (4E-18)	-2.5	-8.3	-38.6	-23.4	-15.0	-17.6	-16.6
MGG_10192.6	Eukaryotic translation initiation factor 3 sub A	-2.4	-2.7	-3.4	-3.6	-3.5	-3.2	-3.3
MGG_15047.6	Major facilitator superfamily transporter	-2.4	-7.9	-17.6	-21.5	-6.3	-12.2	-18.6
MGG_09373.6	CCCH zinc finger DNA binding protein	-2.4	-3.8	-5.0	-7.1	-4.8	-7.2	-6.3
MGG_12949.6	GTP-binding protein 1	-2.4	-5.4	-11.0	-10.0	-11.1	-7.6	-10.1
MGG_00521.6	GTP-binding protein (0.0)	-2.4	-3.8	-2.9	-2.4	-2.9	-2.2	-3.4
MGG_10037.6	Unknown	-2.4	-4.5	-24.4	-16.8	-27.0	-17.9	-25.4
MGG_15066.6	Unknown	-2.4	-6.9	-11.1	-4.8	-6.6	-3.6	-7.8
MGG_15144.6	Unknown	-2.4	-5.8	-3.9	-9.0	-2.2	-3.3	-5.4
MGG_15324.6	Unknown	-2.4	-5.7	-3.4	-4.7	-2.2	-2.4	-5.9
MGG_05749.6	Unknown	-2.4	-5.1	-10.7	-14.5	-5.1	-6.7	-9.8
MGG_00369.6	Cell division cycle protein 123	-2.3	-3.3	-7.3	-6.1	-7.1	-2.6	-5.4
MGG_01453.6	Serine peptidase (3E-113)	-2.3	-3.1	-5.6	-6.3	-2.9	-2.5	-2.5
MGG_06463.6	Unknown	-2.3	-5.8	-6.0	-6.8	-3.8	-5.3	-10.0
MGG_04609.6	Unknown	-2.3	-4.5	-4.3	-4.1	-4.1	-4.3	-6.5
MGG_04058.6	Oxidoreductase/dehydrogenase (3E-151)	-2.3	-2.6	-2.9	-3.5	-2.6	-2.9	-3.1
MGG_02893.6	Integral membrane protein (1E-04)	-2.3	-2.8	-4.2	-2.1	-3.9	-3.4	-5.5
MGG_05987.6	Unknown	-2.3	-3.6	-6.4	-7.8	-5.6	-6.1	-7.5
MGG_01826.6	Cell cycle control protein (Cwf26) (2E-57)	-2.3	-3.1	-4.7	-5.4	-4.4	-2.2	-2.9
MGG_07284.6	WD repeat-containing protein pop3	-2.2	-4.8	-10.3	-10.6	-9.5	-6.1	-6.6
MGG_05322.6	Zinc finger protein 467	-2.2	-4.9	-3.5	-4.3	-3.5	-3.3	-5.1
MGG_08582.6	6-phosphofructo-2-kinase 1	-2.2	-2.9	-3.8	-3.3	-4.1	-4.6	-2.9
MGG_08054.6	Chitinase 1	-2.2	-3.5	-2.3	-2.1	-2.3	-2.6	-4.1
MGG_01087.6	Ribosome biogenesis protein TSR1	-2.2	-2.8	-4.5	-4.4	-4.0	-3.4	-3.5
MGG_05748.6	Unknown	-2.2	-4.1	-4.6	-8.1	-4.8	-6.8	-6.0
MGG_13582.6	Unknown	-2.2	-4.9	-4.3	-4.5	-3.5	-7.9	-11.1
MGG_15292.6	V-type ATPase, C subfamily protein (1E-17)	-2.2	-5.3	-17.6	-16.4	-8.2	-8.1	-14.4
MGG_00893.6	Pfs and NB-ARC domain-containing protein	-2.1	-4.4	-6.3	-4.6	-2.9	-3.3	-3.5
MGG_03493.6	Unknown	-2.1	-3.6	-9.9	-7.9	-5.7	-2.9	-6.4
MGG_04959.6	Unknown	-2.1	-3.6	-7.5	-6.9	-5.7	-3.3	-6.8
MGG_15343.6	Unknown	-2.1	-3.6	-6.3	-6.0	-4.5	-3.3	-5.0
MGG_07089.6	Poly(A) polymerase	-2.0	-3.7	-3.3	-2.7	-3.2	-2.3	-2.7
MGG_05432.6	Asparaginyl-tRNA synthetase	-2.0	-3.4	-3.5	-3.3	-2.4	-2.6	-4.2
MGG_08133.6	Pre-mRNA-processing factor 17	-2.0	-2.7	-6.6	-7.0	-6.5	-5.3	-6.2

^1^list sorted in ascending fold-change in the rice treatment. R: rice at 72 hpi; B: barley at 72 hpi; TS: temperature up shift, PQ: Paraquat, MM: minimal medium, MM-C: carbon limitation, MM-N: nitrogen limitation. The adjusted P-values for these genes are < 0.01.

Among the repressed genes was a methyltransferase (MGG_07911.6), a sugar transporter (MGG_09827.6), an acetyltransferase (MGG_03416.6), two putative *MFS *transporters (MGG_15047.6 and MGG_00419.6), a secretory phospholipase A2 (MGG_09019.6), and a putative linoleate diol synthase (*LDS*; MGG_10859.6).

**Figure 3:**
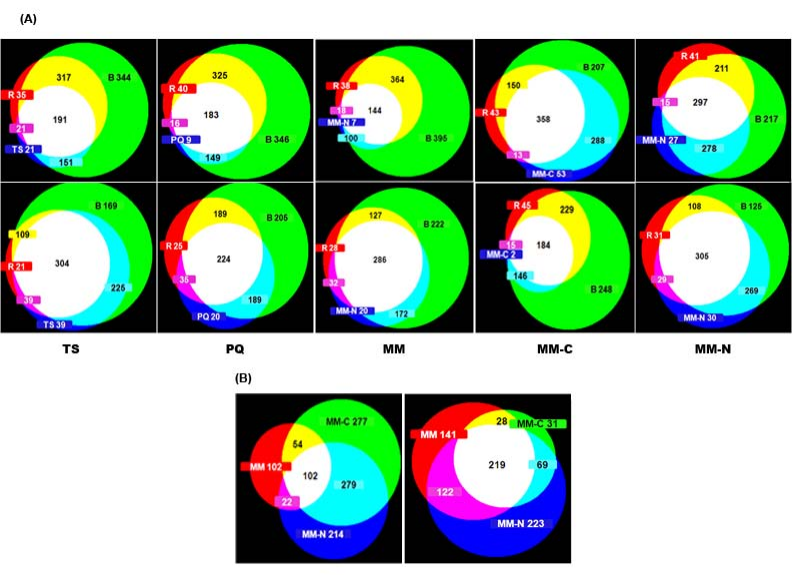
**Venn diagrams of *M. oryzae *genes differentially expressed in each of the *in vitro *treatments compared to *M. oryzae *inoculated in rice and barley leaves**. (A) Number of commonly induced (*upper panel*) or repressed (*lower panel*) fungal genes during barley and rice infection, with each individual *in vitro *condition. Temperature up shift (TS); paraquat (PQ); minimal medium (MM); carbon-limited minimal medium (MM-C); nitrogen-limited minimal medium (MM-N). (B) Number of commonly induced (*left panel*) and repressed (*right panel*) fungal genes in the nutrient-limited conditions.

In order to examine overlapping gene expression profiles between *in planta *expression and individual *in vitro *stresses, we generated area-proportional Venn diagrams of induced and repressed genes for rice, barley and each of the *in vitro *treatments (Figure 3A), along with diagrams showing overlap among the nutrient-limited conditions (Figure 3B).

### *M. oryzae genes *expressed *in planta *and in temperature up shift condition

The temperature up shift condition was included for two reasons: first, we wished to explore the molecular mechanisms that *M. oryzae *uses to adjust to a rapid change in temperature and second, we wished to determine whether any of these mechanisms were similar to those exploited *in planta*. Thus, the list containing the 4,973 differentially expressed genes was sorted for genes that were either induced or repressed in rice, barley, and TS. This resulted in the identification of 191 commonly induced and 304 commonly repressed genes (Figure 3A-first panel). From those 191 induced genes, 121 genes have known functions and among them were several stress-related genes, such as a stress-responsive gene (MGG_05763.6), a superoxide dismutase (MGG_07697.6), a glutathione-dependent redox enzyme (MGG_05447.6), and a glucose-6-phosphate dehydrogenase *G6PD *(MGG_09926.6). The three latter enzymes are known to be involved in antioxidant defense in yeast [34].

Out of the 304 repressed genes, only 127 genes have known functions and among them were a pH-response regulator (MGG_06440.6), a translational regulator (MGG_13245.6), a transcriptional regulator *HAC1 *(MGG_09010.6), a manganese resistance gene (MGG_09884.6), and a cell division cycle gene (MGG_00369.6). Remarkably, no putative heat shock or chaperone-related genes were present in the 191 induced and 304 repressed gene lists, which might be due to the need for higher heat shock temperatures or their differential expression might occur at a time point different than the one selected for this experiment.

### *M. oryzae genes *expressed *in planta *and in Paraquat-induced oxidative stress

From the 4,973 differentially expressed genes, we identified 183 induced and 224 repressed genes in rice, barley, and PQ treatments (Figure 3A- second panel). From those 183 genes, 115 genes have known functions. Notably, one of the induced genes was a glutathione S-transferase (MGG_09138.6), which was also induced in MM-C and MM-N. Additional noteworthy genes were a peroxiredoxin type-2 gene (MGG_02710.6), a regulatory protein ALCR (MGG_02129.6), a polyketide synthase (MGG_14897.6), and a norsolorinic acid reductase homologue (MGG_01713.6). Norsolorinic acid reductase is a polyketide precursor of the aflatoxin B1, which is produced in certain strains of *Aspergillus flavus *and *A. parasiticus *[35].

Among the repressed genes *in planta *and in PQ was a general amino acid permease *GAP1 *(MGG_07606.6), a homologue of *S. cerevisiae *which was shown to be involved in the uptake of the proline analogue azetidine-2-carboxylate, which can be incorporated into proteins competitively with proline and can misfold proteins and be toxic to the cells [36,37]. A GTP-binding protein YPT3 (MGG_07191.6) and a protease ULP1 (MGG_03144.6) were also present in the dataset. The yeast ULP1 protein was reported to be involved in maintenance of unspliced mRNA in the nucleus [38]. However, to our knowledge the additional role of this vital mechanism during fungal invasive growth has not been addressed.

### *M. oryzae genes *expressed *in planta *and in nutrient limitation

From the original set of 4,973 differentially expressed genes, we sorted the data for genes that exhibited common expression patterns between rice, barley, and one of the three nutritional conditions: minimal medium (MM), carbon-limited minimal medium (MM-C), and nitrogen-limited minimal medium (MM-N).

#### Genes expressed in R, B, and MM

There were 144 induced and 286 repressed genes among rice, barley, and MM (Figure 3A- third panel). From the 144 induced genes, 89 have known functions; two of which were a zinc metalloprotease (MGG_10104.6) and a high affinity copper transporter (MGG_07832.6). Among the 286 repressed genes, only 121 have known functions. Interestingly, a DNA damage response protein kinase *DUN1 *gene (MGG_01596.6), a UDP-N-acetylglucosamine transporter *YEA4 *(MGG_05631.6), a GTPase-activating *GYP7 *(MGG_04067.6), and a GTP-binding *YPT3 *(MGG_07191.6) gene were down-regulated. In yeast, the GYP7 protein activates the Rab family of proteins, of which YPT3 is a member, and this protein is involved in vesicle-mediated protein trafficking in exocytosis and endocytosis [39].

#### Genes expressed in rice, barley, and MM-C

The rice, barley, and MM-C treatments revealed 358 induced and 184 repressed genes, from which 220 and 83 genes have known functions, respectively (Figure 3A- fourth panel). Among the induced genes, there were several transporters including a lactose permease (MGG_05889.6), a quinate permease (MGG_08937.6) and three maltose permease *MAL31*genes (MGG_05941.6, MGG_07844.6, and MGG_09607.6). Several other noteworthy up-regulated genes included, a peroxisomal 2,4-dienoyl-CoA reductase *SPS19 *(MGG_05138.6), an aquaporin-9 *AQY2 *(MGG_13615.6), a glycerol kinase *GUT1 *(MGG_10005.6), and a sorbose reductase (MGG_07883.6). In yeast cells, *AQY2 *is correlated with freeze tolerance [40] and *GUT1*is induced under several stress conditions [41]. Cytochrome P450 (CYP) genes are known to be involved in fungal adaptation to new niches, which would likely include overcoming stressful environments [42]. We found three CYP's in our dataset (MGG_04345.6, MGG_09920.6, and MGG_07406.6), which showed the same general trend of having increased expression in barley along with the MM-C conditions. We further observed that an aldehyde dehydrogenase gene (MGG_03900.6) was among the list of induced genes in all treatments and that an alcohol oxidase gene (MGG_09072.6) was highly induced in rice, barley, and MM-C.

Among the repressed genes in rice, barley, and MM-C were a glutamine synthetase *GLN1 *(MGG_14279.6), a magnesium transporter *ALR2 *(MGG_08843.6), and a putative acetolacetate synthase ILV2 (MGG_01104.6). The latter is involved in isoleucine and valine biosynthesis and has been of interest as a target for antifungal products [43].

#### Genes expressed in rice, barley, and MM-N

There were 297 induced and 305 repressed genes, from which 172 and 132 genes have known functions, respectively (Figure 3A- fifth panel), among the rice, barley and MM-N conditions. Among the induced genes, there was a glucosamine 6-phosphate N-acetyltransferase *GNA1 *(MGG_02834.6), a vacuolar aminopeptidase *LAP4 *(MGG_07536.6), a mitochondrial peroxiredoxin *PRX1 *(MGG_08256.6), and a general amino acid permease *AGP2 *(MGG_13334.6). Another general amino acid permease, *AGP3 *(MGG_05107.6), was induced only in MM-N.

In our dataset, a pectate lyase gene (MGG_05875.6) was induced in rice, barley, and MM-N, and very slightly induced in MM-C (1.6 fold). Another pectate lyase (MGG_07566.6) was only induced in barley. Fungal pectate lyases (PELs) are involved in plant cell wall degradation. In necrotrophic pathogens, PELs have been shown to be required for partial or full virulence, whereas biotrophic pathogens have fewer cell wall degrading enzymes (CWDE; reviewed in [44]). Among the repressed genes, there was a NAD-dependent deacetylase sirtuin 5 (MGG_15048.6), an arrestin (MGG_05030.6), and an aspartate aminotransferase *AAT2 *(MGG_04156.6). The *M. oryzae *gene *MPG1 *(MGG_10315.6), encoding a hydrophobin protein, was induced in rice, barley, MM-C, and MM-N treatments as previously observed by Talbot and colleagues [45].

### *M. oryzae genes *expressed only in Rice and/or in Barley

We identified 853 and 376 genes that were specifically induced or repressed, respectively, in rice and/or barley. From these sets, 271 induced and 110 repressed genes were shared by rice and barley. Among the 271 induced genes there were several CWDEs, putative secreted proteins, and different types of membrane transporters. We also observed that a *MAS3 *gene (MGG_00703.6), a cutinase gene (MGG_09100.6), an endoglucanase gene (MGG_14954.6), and an exoglucanase gene (MGG_05520.6) were induced. Three out of the four biotrophy-associated secreted [*BAS-1 *(MGG_04795.6), *BAS-2 *(MGG_09693.6), and *BAS-4 *(MGG_10914.6)] genes identified by Mosquera and colleagues [10] at 36 hours post-inoculation were among the genes only induced during *in planta *infection of rice and barley. Five membrane transporters were present in the list of induced genes, which were an ammonium transporter (MGG_04576.6), an organic anion transporter (MGG_05009.6), a malic acid transporter (MGG_09085.6), an *ABC*-type Fe^3+ ^transporter (MGG_10060.6), and an ABC transporter *CDR4 *(MGG_07375.6). Two other genes, an alcohol (MGG_05519.6) and an aldehyde dehydrogenase (MGG_05008.6) were induced only in rice and barley, which further suggests the importance of such classes of genes for invasive growth.

Among the repressed genes there was a multidrug and toxin extrusion gene (MGG_10534.6), a serine/threonine protein kinase *SAPK1 *gene (MGG_06070.6), and a mitochondrial Rho *GTPase 1 *gene (MGG_01044.6).

### Expression patterns of known pathogenicity genes

**Figure 4:**
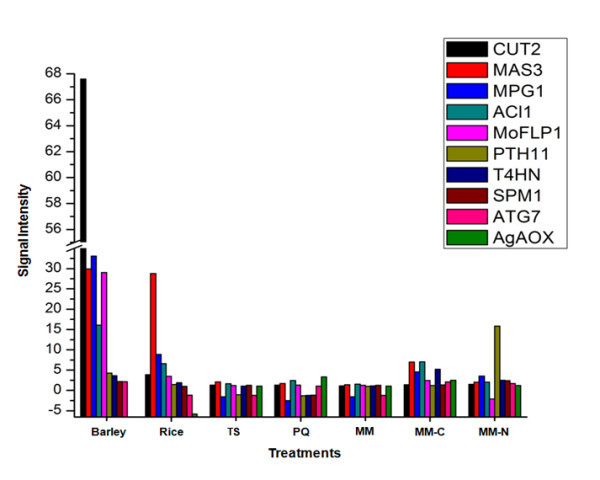
**Gene expression profiles of known pathogenicity genes in *M. oryzae *during *in vitro *and *in planta *treatments**. Induction/repression of known pathogenicity genes in *M. oryzae *subjected to *in vitro *stresses (TS, PQ, MM, MM-C, and MM-N) or inoculated in rice (R) or barley (B) leaves. Literature reference for each gene: *MAS3 *(Mathioni and Donofrio, unpublished); *MPG1 *[45]; *ACI1 *[60]; *CUT2 *[47]*MoFLP1*[48]; *T4HN *reductase [61]; *PTH11 *[62]; *SPM1 *[7]; *ATG7 *[63]; *MgAOX *[64].

We examined the data set for known pathogenicity genes. Given that we chose a time-point more indicative of invasive growth (72 hpi), we did not expect to find many genes solely associated with pre-penetration and penetration since these events are complete by 24 hpi. However, nine pathogenicity genes were identified with common expression profiles in rice or barley and at least one other condition (Figure 4). All of the genes, with the exception of the *MgAOX *gene (MGG_12936.6), which encodes an alternative oxidase and is only induced during the *in vitro *conditions, were induced in one or both plant hosts along with MM-C and/or MM-N. The expression profiles for *MPG1 *(MGG_10315.6) and *SPM1 *(MGG_03670.6) support their previous roles in both virulence and growth during nitrogen limitation [7,17]. *CUT2 *(MGG_09100.6) was the only pathogenicity gene that is uniquely induced during *in planta *growth in both rice and barley at 72 hpi.

### Expression patterns of cell wall degrading enzymes

**Figure 5:**
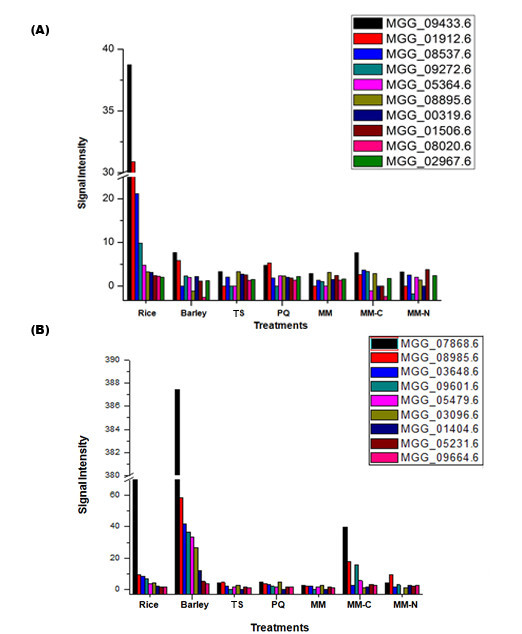
**Response of genes with putative roles in plant cell wall degradation**. Induction/repression of fungal genes subjected to *in vitro *and *in planta *stresses. (A) The expression patterns of ten genes with putative roles in cellulose degradation; (B) the expression patterns of nine genes with putative roles in xylan degradation.

Our data strongly suggested that at 72 hpi, *M. oryzae *activates a cadre of plant CWDEs. This group of enzymes may play a role in fungal cell to cell movement and/or utilize the breakdown products as a nutrient source. From our list of 1,909 genes, we identified ten genes predicted to be involved in xylan breakdown and ten in cellulose breakdown, and examined their expression patterns. With the exception of two genes (MMG_08985.6 and MGG_08020.6), all had induced expression in at least one of the plant hosts (Figure 5) and in at least one of the *in vitro *conditions. Of the predicted cellulose degradation genes, a neutral α-glucosidase had increased expression in all conditions (MGG_09433.6; Figure 5A). Of the predicted xylanase genes, two had increased expression in every condition, an endo-1,4-β xylanase (MGG_07868.6) and a β-xylosidase (MGG_08985.6; Figure 5B). Interestingly, MGG_07868.6 was the most highly expressed gene in barley, and the fifth most highly expressed gene in rice. While it is difficult to predict what their role might be during the *in vitro *growth conditions, high expression of the putative cellulases and xylanases *in planta *at 72 hpi strongly implies that *M. oryzae *is breaking down plant cell walls during invasive growth, which will need further scientific confirmation.

### Time-course of gene expression in barley

**Figure 6:**
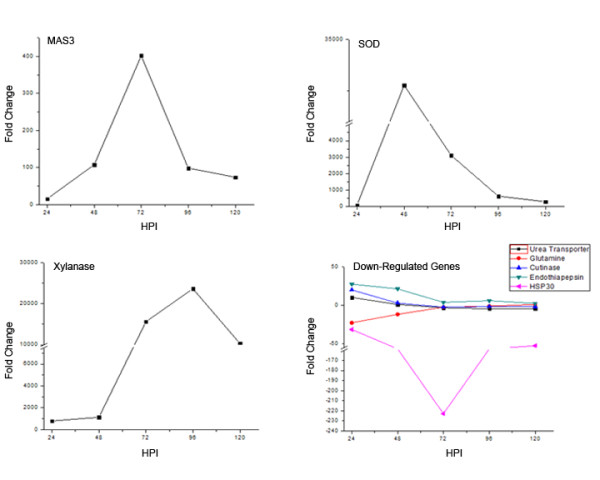
**Gene profiling during a barley infection time-course**. Eight genes were chosen from the microarray validation experiments with increased gene expression among all seven conditions (MAS3: MGG_09875.6; SOD: MGG_07697.6; xylanase: MGG_07868.6) and decreased gene expression among all seven conditions (cutinase: MGG_02393.6; endothiapepsin: MGG_02201.6; *HSP30*: MGG_05719.6; urea active transporter: MGG_09063.6; glutamine synthetase: MGG_06888.6), compared to the reference sample. Real-time qRT-PCR was performed on all eight genes, with glyceraldehyde 3-phosphate dehydrogenase (GAPDH) used as the endogenous control (housekeeping gene). Five time-points were examined, 24, 48, 72, 96 and 120 hours post-inoculation (hpi), and all samples were performed in triplicate.

In order to further characterize genes from our microarray dataset, we chose a subset of genes from Additional file 2 (validation of microarray results) for profiling during a time-course of infection on barley. We chose three genes that were induced in all conditions, *MAS3 *(CAS1 domain-containing protein; MGG_09875.6), *SOD *(superoxide dismutase; MGG_07697.6) and a xylanase gene (MGG_07868.6). We also chose five genes that were repressed in all conditions, endothiapepsin (MGG_02201.6), *Hsp30 *(MGG_05719.6), urea active transporter (MGG_09063.6), glutamine synthetase (MGG_06888.6) and cutinase (MGG_02393.6). All eight genes were profiled using real-time qRT-PCR, with GAPDH as the endogenous control (Figure 6). Interestingly, all five of these genes that were down-regulated in most conditions, were also down-regulated during the 120 hour time-course on barley; the exceptions were cutinase and endothiapepsin, which both showed induction at 24 hpi, but then fell off by the 48 hour time point. Each of the three induced genes peaked at a different time-point; *MAS3 *showed the highest level of expression at 72 hpi, *SOD *at 48 hpi and xylanase at 96 hpi. The fold changes of all three genes matched their microarray profiles for the barley condition, providing yet another confirmation of our microarray dataset.

## Discussion

We hypothesized that the rice blast fungus must be able to grow in, and overcome, an inhospitable environment during invasive growth (72 hpi), including foraging for nutrients and coping with plant defense responses. More specifically, we anticipated observing clustering of gene expression predominantly among the *in planta *conditions (rice and barley), the nutrient deprived conditions (MM-C and MM-N) and the reactive oxygen species condition (PQ). The hierarchical clustering analysis revealed that our hypothesis was at least partially correct in that the strongest relationship between gene expression in rice and barley was with gene expression in MM-C and MM-N. The PQ condition was more closely related to the MM and TS conditions, and the latter two were the most closely related to each other. Overall, these findings indicated that *M. oryzae *is likely coping with limited nutrient availability during the invasive growth stage (72 hpi), and less so with host defense responses (e.g. ROS), or a condition analogous to temperature up shift. It is possible that genes involved in oxidative stress would respond more similarly to *in planta *infection if they were sampled at a different time point. Similarly, a temperature shift may more likely occur earlier in the infection cycle upon the pathogen first entering the leaf. We were surprised to find no significantly induced heat shock or chaperone-like genes induced during this condition. However, a similar experiment was recently performed in *M. oryzae *to examine expression of two Hsp70 genes; the authors examined transcript levels at the same temperature we used, 42°C, across 5, 10, 20 and 40 minutes. Gene levels did not change appreciably from the control samples however one gene's expression decreased when the fungus was exposed to 42°C for longer periods of time [22]. Of the thirteen predicted Hsp70 family members in *M. oryzae*, we found three with increased expression in our nutrient-limited or barley dataset (MGG_06065.6, MGG_09631.6 and MGG_02503.6), but not in our temperature up-shift dataset. Interestingly, MGG_02503.6, which is the Kar2p yeast homolog, is slightly induced during infection of barley and from the study mentioned above and appears to be an essential gene in this fungus [22]. Together, these data suggest the hypothesis that it takes *M. oryzae *a longer period of time to adjust to the heat shock than we had anticipated and that some Hsp's likely play a role in nutrient-limitation and *in planta *growth, as opposed to increased temperatures.

We cannot rule out the possibility that we did not choose the time-points and conditions most similar to the *in planta *situation, or that the ones we did choose may not be more similar to earlier infection time-points rather than to 72 hours post-inoculation. Further, we recognize that had different time-points been chosen for each treatment, we may have discovered additional gene expression patterns. However for the purposes of this study, we wished to examine what the fungus is likely faced with during invasive growth, a stage which remains a black box in *M. oryzae *pathology. Our results lay the groundwork for formulating many testable hypotheses on how this fungus manages stressful environments.

Out of 4,793 differentially expressed genes, 55 and 129 were induced and repressed in all seven conditions, respectively. One of our most striking results was that most genes within the collection of 55 are enzymes with predicted roles in cell wall degradation and carbon metabolism (Figure 3). Furthermore, fungal gene expression in rice and barley groups closely with fungal gene expression during carbon and nitrogen limiting conditions. Together, these results suggest that *M. oryzae *at 72 hpi is nutrient deprived and may be degrading plant cell walls as a usable carbon source.

The induction of many cell wall degrading enzymes (CWDE), observed in the present study, may also suggest that at 72 hpi *M. oryzae *is transitioning from the biotrophic to the necrotrophic phase of the disease cycle. Destruction of plant cell walls likely leads to host cell death. The induction of several CWDEs, such as PEL (MGG_05875.6), in the MM-N condition is not well understood and further studies are required to elucidate their role during nitrogen limitation. Necrotrophic pathogens produce many CWDE including PELs. Several CWDEs were shown to be involved in pathogenesis. For example, a double disruption of two of the four pectate lyases (PEL) present in the pea hemibiotroph pathogen *Nectria haematococca *genome rendered mutants drastically reduced in virulence [46], but single gene knockouts did not affect virulence. Such findings suggest that in highly redundant gene families, such as PELs, there is a need for creating multiple combined knockouts.

Most genes known to play a role in pathogenicity in the rice blast fungus are involved in pre-penetration or penetration stages. Therefore, we did not expect to encounter many known pathogenicity genes in our *in planta *dataset for rice and barley. However, several genes that are known to play a role in either invasive growth, or in multiple stages of the disease cycle, were detected in our microarray studies. Such genes included *MPG1 *(MGG_10315.6) and cutinase *CUT2 *(MGG_09100.6). The hydrophobin *MPG1 *gene (MGG_10315.6), which was first identified in cDNA libraries from invasive growth of *M. oryzae *and shown to be induced during *in planta *growth as well as under *in vitro *carbon and nitrogen limitation conditions [45], was also induced in our experiments in R, B, MM-C, and MM-N conditions. From previous studies, the *CUT2 *gene was shown to be highly induced during appressorium maturation and penetration in *M. oryzae *and the *cut2 *mutant showed a reduced pathogenicity phenotype (smaller and fewer lesions) compared to wild type, [32]. It was also highly induced at 48 hpi in that same study. The strong induction of this gene in the present study at 72 hpi suggests it might play a role during *in planta *invasive hyphal growth and during conidiophore emergence through the cuticle, as previously proposed by Sweigard and colleagues [47]. However, further investigation is required to elucidate its role during the invasive infection stage in the host plant.

Additionally, induction of the biotrophy-associated secreted (BAS) genes identified by Mosquera and colleagues [10] at 36 hpi supports our findings. Three out of the four BAS genes [BAS-1 (MGG_04795.6), BAS-2 (MGG_09693.6), and BAS-4 (MGG_10914.6)] were among the genes only induced during *in planta *infection of rice and barley. We found it notable that of the pathogenicity genes examined, *CUT2, MAS3, ACI1 *and *MoFLP1 *were more strongly expressed in barley than in rice (Figure 4). Had all the pathogenicity genes had a higher fold change in barley than rice, we might have attributed it simply to increased fungal biomass; the *M. oryzae *strain we used appears to colonize barley roughly 8-12 hours faster than rice. However, since only four of the ten genes examined had this molecular phenotype, we hypothesize that they must be more necessary in barley than rice for invasive growth. We are currently testing this hypothesis with the MAS3 gene by deleting it from two strains, one that only infects barley and another that infects both rice and barley. The *MoFLP1 *gene is a member of the fasciclin family and predicted to be involved in cell adhesion; null mutants in this gene render the fungus less adhesive and less pathogenic [48]. It is difficult to speculate why this transcript is about 10-fold higher in barley versus rice, except that perhaps the cell wall and/or wax structure of barley leaves may differ enough from rice, such that more of this gene product needs to be produced in order to attach sufficiently to barley. Likewise, the *CUT2 *gene shows about a 17-fold increase while infecting barley, versus rice. This was a surprising result, considering we expect cutinase to be expressed more strongly during initial penetration events, rather than later in infection. One possibility is that because *M. oryzae *tends to infect barley more rapidly than rice, by 72 hpi it might need to be producing copious amounts of cutinase to forge its way out through the plant's cuticle. The cutinase family has been previously examined in *M. oryzae*, and consists of 17 members [49]. *CUT2 *showed strongest expression during penetration, about 12 hpi and dropped off by 48 hpi, however in their transcriptional profiling of 14 of the 17 members, the authors did not go further than 48 hpi. We did examine the expression of one cutinase family member (MGG_02393.6) across a time-course of infection in barley, and found that this gene started off strongest at 24 hpi, and fell sharply by 48 hpi. Interestingly, Skamnioti and colleagues (2008) considered this gene to be "constitutively expressed", showing induction at some pre-penetration and penetration time-points, but then falling off by 48 hpi, which is in accordance with our result. Taken together, our data and previously published data on the cutinase family members indicate that individual genes play different roles at different times during infection; localization experiments during infection time-courses would certainly help to elucidate when they are required.

Our barley infection time-course also provided insight into a relatively unknown gene in *M. oryzae*, the *MAS3 *(CAS1 domain-containing; MGG_09875.6) gene. This seven member family of proteins in *M. oryzae *has been studied with respect to their role in appressorial formation, and two members have been deleted from a related strain to 70-15, Guy11 [6,50]. These two members played a role in pathogenicity, but the gene studied here, MAS3, has not yet been looked at with regard to either of these important disease cycle stages. Oh and colleagues [6] noted that MAS3 was down-regulated during appressorial development, while another member of the family, MGG_12337.6, was strongly up-regulated. Conversely, we have discovered that MAS3 is highly up-regulated during invasive growth, while MGG_12337.6 is strongly down-regulated. Here, we find another case of individual family members playing roles at specific times during the infection cycle.

In the present study, we observed a high affinity amino acid permease *AGP2 *(MGG_13334.6) and an amino acid permease *GAP1 *(MGG_07606.6) among the differentially expressed genes. *AGP2 *(MGG_13334.6) was highly expressed in rice, barley, MM-C, and MM-N. Higher fold changes were observed in rice and barley (18.5 and 20.4, respectively), compared with 3.8 and 4.4 in MM-C and MM-N, respectively. On the other hand, the rice blast *GAP1 *gene showed repression in rice and barley (-3.4 and -2.8, respectively), and a moderated induction in MM-C and MM-N (2.1 and 2.9, respectively). In *S. cerevisiae*, *AGP2 *was shown to be highly involved in amino acid uptake in poor nitrogen conditions and its expression level was increased in the absence of GAP1[51]. Thus, the observation that *AGP2 *is highly expressed whereas *GAP1 *is down-regulated is similar to that observed by Schreve and Garrets [51], and allows us to speculate that *AGP2 *has to compensate the absence of *GAP1 *when it is repressed, but when it is moderately induced, *AGP2 *is also moderately induced. Further studies are necessary to verify whether the co-regulation of such genes occurs and its significance to fungal invasive growth.

The oxidative stress induces the production of aflatoxin B1 and its precursors, such as norsolorinic acid as it has been demonstrated in *A. parasiticus *[35]. A norsolorinic acid reductase homologue (MGG_01713.6; induced in rice, barley, PQ, and MM-C) and two other putative aflatoxin precursor homologues were also present in our differentially expressed dataset, which are a sterigmatocystin 8-O-methyltransferase precursor (MGG_02120.6; induced in rice, barley, PQ, and MM-N) and a versicolorin reductase (MGG_07216.6; induced in all treatments except PQ). However, BLAST analysis showed that MGG_01713.6 is highly conserved in fungi (data not shown) including those that are not known to produce aflatoxin, suggesting it might have other functions. Interestingly, a NADP-dependent mannitol dehydrogenase (MGG_06779.6) was highly induced in rice, barley, TS, PQ, and MM. This enzyme has been suggested to be involved in oxidative protection in several plant fungi but the exact mechanism of protection has not yet been determined [52].

ABC transporters are known to play a role in fungal cells by diminishing toxic compounds produced by the plant cells. One *M. oryzae *ABC transporter, named *ABC1 *(AF032443; 100% similarity to MGG_13624.6), has been characterized and deletion mutants showed a decrease in growth and died shortly after penetration of either rice or barley epidermal cells [53]. In our specific expression dataset, the *ABC *transporter (MGG_13624.6) was significantly repressed in R and in all stress treatments, which suggests that this gene might be involved in other stages of infection of *M. oryzae*. We also speculate that the function of this gene might be carried out more efficiently by other members of this large gene family.

With the expression data we have collected from several environmental conditions, we are in a position to begin to identify interaction networks. To investigate possible interactions we selected two genes that were induced under all seven conditions when compared to the reference sample. We then used these genes to perform a network analysis with the commercially available software IPA (Ingenuity Pathway Analysis; see Methods for details).

**Figure 7:**
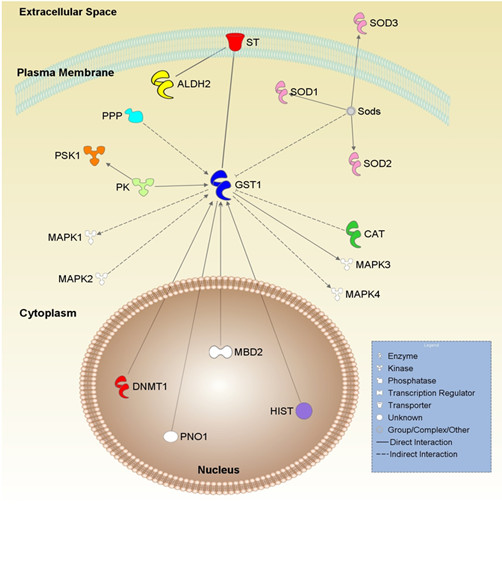
**Glutathione-S-transferase interacting network**. We utilized the IPA software to develop an interaction pathway around a gene, The Glutathione-S-transferase (MGG_05565.6), induced during all seven conditions. The GST interacts with several genes involved in reactive oxygen species scavenging, as well as with MAP kinase signal transduction pathways. Abbreviations are as follows: *GST *= glutathione-S-transferase; *SOD *= superoxide dismutase; *MAPK *= MAP kinase; *DNMT1 *= DNA methyltransferase 1; *CAT *= catalase; *ALDH2 *= aldehyde dehydrogenase; *ST *= sugar transporter; *HIST *= histone deacetylase and methyltransferase; *PNO1 *= phosphomannosylation of N-linked oligosaccharides; PSK1 = protein kinase; PK = protein kinase; PPP = protein phosphatase; MBD2 = methyl CpG binding domain 2.

Our selected genes for network analysis were an aldehyde dehydrogenase (*ALDH*; MGG_03900.6) and a glutathione S-transferase (*GST*; MGG_05565.6), both of which showed increased expression in all seven conditions. Along with their expression profiles, they were chosen because of previous work that showed induction during interactions between the fungal apoplastic pathogen *Cladosporium fulvum *and tomato (aldehyde dehydrogenase), and their role in detoxification of reactive oxygen species (GST), respectively [54,55]. Coleman and colleagues [54] reported an aldehyde dehydrogenase gene being induced during *in planta *growth and *in vitro *carbon and nitrogen starvation in *C. fulvum*, a non-obligate biotroph pathogen found on tomato. They suggested that starvation is likely one of the environmental signals for the expression of genes required for fungal growth in plant hosts. These genes have homologues in *S. cerevisiae*, and were used to create a network and the interacting molecules are shown (Figure 7). Based on the interaction networks found in IPA, the ALDH and the GST directly regulate a sugar transporter (ST). The superoxide dismutases and a catalase indirectly inhibit GST, whereas GST directly and indirectly regulates several mitogen-activated protein kinases (MAPK), which are involved in signal transduction pathways. DNA methyltransferase (DNMT1) and histone methyltransferase and deacetylase (HIST) directly regulate the GST. Expression data from genes in this interaction pathway are shown in Table 4.

**Table 4:** List of putatively interacting genes

Group	MG number	Gene name	R	B	TS	PQ	MM	MM-C	MM-N
**ALDH2**	MGG_03900.6	Aldehyde dehydrogenase	7.7	23.0	3.2	4.1	3.4	9.1	3.7

**GST1**	MGG_05565.6	Glutathione S-transferase	14.2	29.9	5.5	4.8	3.5	7.9	5.5

	MGG_06747.6	Glutathione S-transferase	6.9	8.0	3.6	3.6	3.1	17.2	2.4
	MGG_09138.6	Glutathione S-transferase II	2.7	52.3	1.4	2.8	1.3	25.0	9.5

**SOD**	MGG_07697.6	Superoxide dismutase	121.5	87.7	4.6	1.8	2.2	1.9	-1.4
	MGG_00212.6	Superoxide dismutase	2.2	1.6	1.8	1.9	1.7	1.4	1.0
	MGG_13177.6	Superoxide dismutase	1.5	3.3	2.9	2.9	2.6	1.7	1.2

**CAT**	MGG_09834.6	Peroxidase/catalase 2	1.2	-1.2	1.9	2.1	1.6	1.0	1.4
	MGG_10061.6	Catalase-1	1.4	-1.8	1.7	2.1	1.7	-1.5	-2.4
	MGG_04337.6	Peroxidase/catalase 2	1.2	7.9	1.2	1.7	1.2	2.6	2.1
	MGG_06442.6	Catalase-3	-1.6	-1.7	-1.0	3.0	-1.1	1.2	1.9

**PPP**	MGG_05207.6	Protein phosphatase 2C	2.1	2.3	1.3	1.4	1.3	1.6	2.4

**DNMT1**	MGG_03526.6	DNA methyltransferase 2	5.6	1.1	4.5	4.2	4.2	1.2	-7.5

**HIST**	MGG_04588.6	Sir2 histone deacetylase Hst4	-1.8	-1.7	-2.7	-2.8	-2.6	-3.4	-2.4
	MGG_05254.6	Hist-lysine N-methyltransferase	-2.7	-3.6	-5.5	-7.5	-5.3	-5.1	-4.6
	MGG_06043.6	Histone deacetylase HOS3	-1.6	-2.0	-3.0	-2.2	-3.3	-2.6	-1.4

**Sugar Transporter**	MGG_09852.6	Sugar transporter STL1	39.0	2.6	1.7	2.1	1.7	-3.0	1.4
	MGG_04780.6	Sugar transporter STL1	22.4	2.5	2.1	2.2	2.0	2.0	3.7
	MGG_08446.6	Sugar transporter	3.0	35.1	1.5	2.3	1.4	6.8	2.2

**PK**	MGG_03207.6	Protein kinase putative	3.0	6.9	-2.0	-1.8	-1.8	-2.1	-2.8

**PSK1**	MGG_01260.6	Serine/threo-protein kinase psk1	-3.0	-4.3	-5.6	-10.2	-5.7	-8.0	-9.9

While it is difficult to speculate how GSTs and aldehyde dehydrogenase might regulate sugar transporters, it is well-known that GSTs interact with both SODs and catalases to detoxify reactive oxygen species [56]. Furthermore, given GSTs' role in detoxification, it might be reasonable to speculate that they trigger signalling pathways, such as those governed by MAP kinases. Further experiments are needed to elucidate whether and how such genes are co-regulated and what kind of relationship indeed occurs in the rice blast fungus during invasive growth and stress conditions.

## Conclusions

We have generated a robust dataset which advances our knowledge of the genes involved in stress response and invasive growth of the rice blast pathogen *M. oryzae*. Invasive growth at 72 hpi appears to require genes involved in nutrient acquisition, which is in support of our original hypothesis, and in keeping with previous findings. Future studies aimed at the functional characterization of genes reported here via targeted deletions or further real-time qRT-PCR experiments will help to better define pathways involved in *M. oryzae *invasive growth as well as their importance in earlier and later stages of disease. Further, we demonstrated the utility of our dataset for formulating hypotheses for future research, when paired with literature searches and gene network analysis programs, such as IPA.

## Methods

### Strain and growth conditions

*Magnaporthe oryzae *strain 70-15, for which a genome sequence is available, was used in all experiments. Stocks on filter papers stored at -20 ºC were used to start cultures. The fungus was grown on oatmeal-agar medium (50 g/L oatmeal, 15 g/L agar) for 10 days, under continuous light at 25 ºC. Fungal plugs (5 mm) were transferred to flasks containing liquid complete medium (LCM; 10 g/L sucrose, 6 g/L casamino acids, 6 g/L yeast extract, 0.1% (v/v) trace elements) and incubated in a shaker at 27 ºC and 150 rpm in the dark for 4 days. The mycelia were collected using Whatmann filter paper and washed with distilled autoclaved water to remove any traces of the complete media, as per previous protocols [17]. Adding even small amounts of complete media to the *in vitro *conditions, particularly the nutrient-deprived, may not represent a true "switch" from one treatment to another. The mycelia was then divided into equal parts and used as the common reference for microarrays and as the starting material for all *in vitro *experiments (temperature up shift, oxidative, and nutritional limitation). We chose to use 4-day old mycelial growth as a reference because fungal hyphae are still actively growing; the colony has not yet melanised, the media is not yet used up, and new micro-colonies are still forming. The temperature up shift experiment was done by incubating fungal mycelia in LCM in a darkened shaker (150 rpm) at 42 ºC for 45 min. These conditions were selected based upon a combination of preliminary gene expression data, and the literature [22,57]. The oxidative experiment was carried out using fungal mycelia in LCM plus methyl viologen (Paraquat; 5 mM) in a dark shaker (150 rpm) at 27 ºC for 24 h. This concentration was selected based on a preliminary experiment where we evaluated fungal growth on different concentrations (data not shown). Nutritional limitation experiments were performed in minimal medium (MM; 6 g/L NaNO_3_, 0.5 g/L KCl, 0.5 g/L MgSO_4_, 1.5 g/L KH_2_PO_4_, 0.1% (v/v) trace elements, 10 g/L D-glucose), in a darkened shaker (150 rpm) for 16 h at 27 ºC. Nitrogen and carbon limitation treatments were performed in MM without the nitrogen source (NaNO_3_; MM-N) and without the carbon source (D-glucose; MM-C), respectively. The nutritional limitation conditions were selected based on previous experiments from the literature [17]. For the *in planta *experiments, rice (*Oryza sativa *cultivar Maratelli) and barley (*Hordeum vulgare *cultivar Lacey) detached leaves were inoculated with 1 × 10^5 ^spores/mL. At 72 hpi lesions were collected and used for RNA extraction. Mock inoculated leaves were used as control in the *in planta *experiments. All experiments were performed in three biological replicates.

### RNA extractions and quality checking

RNA extraction was performed using Trizol reagent (Sigma Chemical, St. Louis, MO) following manufacturer's instructions. Briefly, fungal mycelia stored at -80 ºC was ground using liquid nitrogen, placed in Trizol, and the final pellet was re-suspended in 50 μl of DEPC-treated water. RNA was isolated from three biological replicates and then pooled for RNA purification. Isolated RNA was purified using the RNeasy Plant Mini Kit (Qiagen Sciences, Valencia, CA). Genomic DNA extraction was performed using Wizard Genomic DNA Purification Kit (Promega Corporation, Madison, WI) according to manufacturer's instructions. A Bioanalyzer (Agilent Technologies, Wilmington, DE) was used to check RNA quality. RNA was extracted from each biological replicate, pooled for a total of one microgram and outsourced for labelling and hybridizations (see next paragraph). Pooled RNA samples have been used successfully by the authors in previous *M. oryzae *microarray experiments on both nitrogen-limitation and appressorial formation [6,17].

### Microarray cRNA labelling, hybridization and scanning

The isolated RNA (as described above) was sent to Cogenics (now Beckman, Coulter Genomics, Morrisville, NC) for labelling, hybridizations and scanning. Briefly, the protocol for the Agilent microarray platform is as follows: RNA samples are labelled with Cy3 and Cy5 fluorescent dyes using a Low RNA Input Fluorescent Linear Amp Kit (Agilent Technologies, Wilmington, DE) following the manufacturer's instructions. The Agilent's *Magnaporthe grisea *2.0 Oligo Microarray slide (product G2519F) was used in this work. Each slide contains four replicated arrays of 60mer oligonucleotide probes. Each array contains 15,170 probes from *M. oryzae *predicted genes and 6,325 rice gene probes. Each of the *M. oryzae *and rice probes are repeated twice in an array, which totals approximately 44,000 probes, or features (44 K) per array.

Dye swap hybridizations of the labelled cRNA samples are typically performed using 0.5 μg of each sample per hybridization following Agilent's protocol. Each treatment was hybridized with the common reference (*M. oryzae *grown in complete medium) sample for 17 h at 60 ºC in the dark. Washes are typically performed at 25 °C as follows: 10 min in wash #1 (6X SSC) two times; 5 min in wash #2 (0.1X SSC) two times. Slides were immediately dried using a nitrogen gun (Fisher, Pittsburgh, PA) and scanned on an Agilent Technologies High Resolution Microarray Scanner with SureScan technology (product G2565AA).

### Microarray data analyses

Microarray analyses were performed with the Bioconductor/R package Limma [23-28]. Files containing signal and background intensities were used as input. Spots with background greater than 100 relative fluorescent units (rfu) were discarded. The quality of the microarray experiments was checked by plotting the background of each channel (Cy3 or Cy5) for each microarray and by calculating pair-wise correlations between the signal intensities of each channel of dye-swaps and/or replications (Additional file 3). Slides with poor correlation or high background were removed from the analysis, and labelling and hybridization were repeated. We applied background correction in each channel using the method "subtract" within Limma. The sample spots in the microarrays were classified according to their hybridization to either *M. oryzae *or rice and only the spots in which *M. oryzae *hybridized were kept for further analysis. This was necessary because we had treatments of *M. oryzae *either inoculated in plant tissue or grown in axenic cultures that resulted in different amounts of *M. oryzae *total RNA between these types of samples. Instead of applying a correction to the amount of *M. oryzae *RNA in each sample with the goal of using the same final amount of fungal RNA, we corrected the intensities of the microarray signal during the normalization steps of the analyses. We reasoned that changing the initial amounts of RNA before labelling or hybridization would create a kinetics problem during microarray processing, while correcting the signal during the normalization and scaling would allow us to systematically adjust the overall intensity signals from every treatment. During the normalization analyzes, we transformed the microarray signal intensities between channels within the same microarray using the "loess" method and scaled the data between microarrays using the method "Aquantile" in the R software package Limma.

To detect differentially expressed genes we used a linear model that compared all treatments against the common reference sample. The P-values for multiple comparisons were corrected using the method "global" and adjusted to control the false-discovery rate at 1% by the method "BH" in Limma [23]. The null hypothesis tested by the contrasts of adjusted P-values was the absence of differential expression in any treatment in relation to the reference. Transcripts were considered differentially expressed if they had an adjusted P-value < 0.01 and a fold-change greater than two. The list of differentially expressed probes was used to create several data subsets for further analyses. Dendrograms were created using one minus the correlation as distance matrix and average hierarchical clustering. The significance of the found clusters was estimated by 10,000 bootstraps. Heatmaps were created using Limma within R, and Venn diagrams were generated using 3Venn Applet software program [58].

### Accession Number

Microarray data has been deposited in the NCBI GEO database (http://www.ncbi.nlm.nih.gov/projects/geo/;[59]); our data can be found under the accession number GSE21908. We will upload our dataset to the MGOS (*Magnaporthe oryzae*-*Oryza sativa *Interaction Database; http://www.mgosdb.org) database, with a direct link to also be added upon revision.

### Microarray validation

Quantitative reverse transcription-polymerase chain reaction (qRT-PCR) was performed using RealMasterMix SYBR ROX (5 PRIME, Gaithersburg, MD; cat. # 2200800), for SYBR Green fluorescence detection on a Realplex2 Mastercycler (Eppendorf, Westbury, NY). All qRT-PCR primers were tested with RT-PCR before their use. The qRT-PCR reactions were performed in a final volume of 20 μL containing 10 μL of 2.5x MasterMix, 0.06 μL of 100 μM of each forward and reverse primers, and 1 μL of cDNA. The reactions occurred at 95 °C for 2 min, followed by 40 cycles of 95 °C for 15 sec, 57 °C for 20 sec, and 68 °C for 25 sec. Relative expression levels were determined by the ΔΔC_T _method based on three technical replicates per sample and using actin (MGG_05587.6) as the endogenous control. All qRT-PCR reactions were repeated at least twice with similar results. The sequences of all primers are shown in Additional file 4. When the expression level of a particular *M. oryzae *gene was higher than the expression level observed in *M. oryzae *grown in complete media, the reference sample, in both the microarray experiments and in the qRT-PCR was considered a match. The percentage of matched genes in relation to the total number of tested genes was calculated for all treatments in relation to the reference sample. Finally, an averaged percentage was calculated based on the percentage of matches of all treatments in relation to the reference sample.

### Timecourse experiment on barley

The eight day-old cotyledons of barley (*Hordeum vulgare *cultivar Lacey) were inoculated with 20 μL of 1 × 10^5 ^spores/mL. Three biological replicates of the blast lesions were collected in a timecourse experiment (24, 48, 72, 96, and 120 hpi), frozen in liquid nitrogen and kept in -80 °C until RNA extraction. Total RNA was extracted as previously described (see RNA extraction and quality checking section). First-strand cDNA was synthesized from total RNA using the GoScript Reverse Transcription System (Promega Corporation, Madison, WI). qRT-PCR was performed on first-strand cDNA using RealMasterMix SYBR ROX (5 PRIME, Gaithersburg, MD; cat. # 2200800), for SYBR Green fluorescence detection on a Realplex2 Mastercycler (Eppendorf, Westbury, NY) as described previously (see Microarray validation section). Three technical replicates were performed for all reactions. Relative expression levels were determined by the ΔΔC_T _method based on three technical replicates per sample and using glyceraldehyde 3-phosphate dehydrogenase (GAPDH; MGG_01084.6) as the endogenous control. The sequences of all primers are shown in Additional file 4.

### Generation of gene network using Ingenuity Pathway Analysis (IPA) software

Aldehyde dehydrogenase and glutathione S-transferase genes were interrogated using the Ingenuity PathwayAnalysis (IPA) and the resulting interacting molecules for each gene were placed in the same network. IPA is a knowledge database for human, mouse, and rat, but it also displays whether the gene has a homolog in *Saccharomyces cerevisae*. Thus, all *M. oryzae *genes used in this network analysis were first searched for homologues in the Saccharomyces genome database (http://www.yeastgenome.org/).

## Authors' contributions

SMM carried out all the experiments. CR helped to perform preliminary microarray hybridizations, and to familiarize SMM with the Agilent platform. SMM and AB analyzed the data and drafted the manuscript. RAD helped to conceive the study. ND conceived the study, participated in its design and coordination and helped to draft the manuscript. All authors reviewed and performed corrections in the draft versions. All authors read and approved the final manuscript.

## Supplementary Material

Additional file 1 **Lists of the ten most induced and ten most repressed *M. oryzae *genes in the *in planta *and *in vitro *stress conditions**. Click here for file

Additional file 2 **Validation of the microarray experiments by quantitative RT-PCR**. Click here for file

Additional file 3 **Table containing the Pearson correlation coefficients for all the dye swap microarray hybridizations**. Click here for file

Additional file 4 **List of primers used in the validation of the microarray results by quantitative RT-PCR**. Click here for file
