# Supplementary material for: Transcriptome profiling of the rice blast fungus during invasive plant infection and in vitro stresses
Source: BMC Genomics. 2011 Jan 19;12:49. doi: 10.1186/1471-2164-12-49 (PMC3037901; doi:10.1186/1471-2164-12-49)
Supplement: Additional file 1 — Lists of the ten most induced and ten most repressed M. oryzae genes in the in planta and in vitro stress conditions. [file 1471-2164-12-49-S1.DOCX]

**Additional file 1.** Lists of the ten most induced and ten most repressed *M. oryzae* genes in each treatment compared to the reference sample and with known function. The adjusted P-values for these genes are < 0.01.

| **R (72hpi) – 10 most induced genes** | | | |
| --- | --- | --- | --- |
| **Rank** | **Gene Name** | **Annotation** | **FC** |
| 6 | MGG_08519.6 | Aflatoxin B1 aldehyde reductase member 3 | 147.23 |
| 8 | MGG_07697.6 | Superoxide dismutase | 121.46 |
| 12 | MGG_08487.6 | Cellobiose dehydrogenase | 102.07 |
| 18 | MGG_00824.6 | Phenylacetone monooxygenase | 87.15 |
| 19 | MGG_07868.6 | Endo-1,4-beta-xylanase | 86.78 |
| 20 | MGG_10005.6 | Glycerol kinase | 84.42 |
| 21 | MGG_13598.6 | Endothiapepsin | 82.90 |
| 22 | MGG_01365.6 | FAD binding domain-containing protein | 81.73 |
| 25 | MGG_07491.6 | Retinol dehydrogenase 13 | 76.13 |
| 29 | MGG_08416.6 | Lipase 1 | 57.12 |
| **R (72hpi) – 10 most repressed genes** | | | |
| **Rank** | **Gene Name** | **Annotation** | **FC** |
| 3 | MGG_09226.6 | Fumarate reductase | -7.65 |
| 6 | MGG_01114.6 | Alcohol dehydrogenase | -6.77 |
| 8 | MGG_05719.6 | 30 kDa heat shock protein | -6.45 |
| 9 | MGG_04999.6 | External NADH-ubiquinone oxidoreductase 1 | -6.45 |
| 11 | MGG_08019.6 | F-box domain-containing protein | -6.19 |
| 12 | MGG_12936.6 | Alternative oxidase | -5.84 |
| 15 | MGG_00312.6 | Glyoxylate reductase | -5.59 |
| 16 | MGG_06062.6 | Nitrate reductase | -5.51 |
| 17 | MGG_03144.6 | Ulp1 protease family protein | -5.40 |
| 24 | MGG_09359.6 | Alcohol dehydrogenase | -5.18 |

| **B (72hpi) – 10 most induced genes** | | | |
| --- | --- | --- | --- |
| **Rank** | **Gene Name** | **Annotation** | **FC** |
| 1 | MGG_07868.6 | Endo-1,4-beta-xylanase | 387.47 |
| 2 | MGG_10005.6 | Glycerol kinase | 186.45 |
| 5 | MGG_07908.6 | Endoglucanase-6B | 177.22 |
| 6 | MGG_10712.6 | Exoglucanase 1 | 170.04 |
| 8 | MGG_07646.6 | Alpha-glucuronidase | 159.18 |
| 10 | MGG_10040.6 | Ferulic acid esterase A | 134.39 |
| 13 | MGG_05941.6 | Maltose permease MAL31 | 128.19 |
| 14 | MGG_10038.6 | Periplasmic beta-glucosidase/beta-xylosidase | 125.63 |
| 16 | MGG_08752.6 | Exopolygalacturonase | 120.51 |
| 17 | MGG_09314.6 | Lipolytic enzyme | 115.28 |
| **B (72hpi) – 10 most repressed genes** | | | |
| **Rank** | **Gene Name** | **Annotation** | **FC** |
| 3 | MGG_00304.6 | Peptide transporter MTD1 | -30.85 |
| 4 | MGG_09226.6 | Fumarate reductase | -26.24 |
| 8 | MGG_02201.6 | Endothiapepsin | -18.33 |
| 10 | MGG_03416.6 | Acetyltransferase | -16.34 |
| 16 | MGG_13455.6 | Mannan endo-1,6-alpha-mannosidase DCW1 | -12.23 |
| 18 | MGG_01114.6 | Alcohol dehydrogenase | -11.50 |
| 22 | MGG_05719.6 | 30 kDa heat shock protein | -11.31 |
| 23 | MGG_10755.6 | Arylesterase/monooxygenase | -10.71 |
| 25 | MGG_01045.6 | Arrestin domain-containing protein | -10.07 |
| 27 | MGG_07417.6 | DUF895 domain membrane protein | -9.86 |

| **TS – 10 most induced genes** | | | |
| --- | --- | --- | --- |
| **Rank** | **Gene Name** | **Annotation** | **FC** |
| 3 | MGG_01041.6 | Cellulose signaling associated protein ENVOY | 12.59 |
| 4 | MGG_08436.6 | Minor extracellular protease vpr | 12.34 |
| 5 | MGG_07973.6 | Surface protein 1 | 12.09 |
| 6 | MGG_03029.6 | 24 kDa metalloproteinase precursor | 11.45 |
| 10 | MGG_09817.6 | Minor extracellular protease vpr | 9.80 |
| 11 | MGG_09352.6 | Minor extracellular protease vpr | 9.70 |
| 13 | MGG_10585.6 | Monooxygenase | 9.26 |
| 15 | MGG_07146.6 | Glycosyl hydrolase | 9.12 |
| 21 | MGG_06494.6 | D-arabinitol 2-dehydrogenase | 8.26 |
| 23 | MGG_03002.6 | Cryptochrome DASH | 8.12 |
| **TS – 10 most repressed genes** | | | |
| **Rank** | **Gene Name** | **Annotation** | **FC** |
| 1 | MGG_02201.6 | Endothiapepsin | -80.51 |
| 3 | MGG_10715.6 | Zinc carboxypeptidase | -71.96 |
| 7 | MGG_00460.6 | Rhamnolipids 3-oxoacyl-[acyl-carrier-protein] reductase | -39.86 |
| 12 | MGG_02235.6 | Oxidoreductase | -37.58 |
| 13 | MGG_07911.6 | Methyltransferase | -36.08 |
| 16 | MGG_00419.6 | Major facilitator superfamily transporter | -30.51 |
| 19 | MGG_10755.6 | Arylesterase/monooxygenase | -28.13 |
| 23 | MGG_09019.6 | Secretory phospholipase A2 | -23.47 |
| 26 | MGG_05055.6 | Alcohol dehydrogenase | -23.23 |
| 32 | MGG_09990.6 | Minor extracellular protease vpr | -19.21 |

| **PQ – 10 most induced genes** | | | | |
| --- | --- | --- | --- | --- |
| **Rank** | **Gene Name** | **Annotation** | | **FC** |
| 1 | MGG_02069.6 | Glyoxalase/bleomycin resistance protein/dioxygenase | | 34.69 |
| 2 | MGG_05912.6 | N-acyl-L-amino acid amidohydrolase | | 27.46 |
| 5 | MGG_08436.6 | Minor extracellular protease vpr | | 16.89 |
| 6 | MGG_02530.6 | Quinate permease | | 14.04 |
| 10 | MGG_10275.6 | Sphingomyelin phosphodiesterase | | 12.12 |
| 12 | MGG_00385.6 | L-aminoadipate-semialdehyde dehydrogenase | | 11.20 |
| 15 | MGG_01041.6 | Cellulose signaling associated protein ENVOY | | 10.35 |
| 16 | MGG_13290.6 | Alpha/beta hydrolase | | 10.26 |
| 19 | MGG_04099.6 | Allantoate permease | | 8.50 |
| 22 | MGG_10585.6 | Monooxygenase | | 8.43 |
| **PQ – 10 most repressed genes** | | | | |
| **Rank** | **Gene Name** | **Annotation** | | **FC** |
| 3 | MGG_02201.6 | Endothiapepsin | -42.36 | |
| 4 | MGG_07911.6 | Methyltransferase | -34.29 | |
| 9 | MGG_00460.6 | Rhamnolipids 3-oxoacyl-[acyl-carrier-protein] reductase | -24.50 | |
| 10 | MGG_02235.6 | Oxidoreductase | -24.36 | |
| 12 | MGG_10755.6 | Arylesterase/monooxygenase | -23.33 | |
| 13 | MGG_09990.6 | Minor extracellular protease vpr | -21.62 | |
| 14 | MGG_15047.6 | Major facilitator superfamily transporter | -21.50 | |
| 16 | MGG_00419.6 | Major facilitator superfamily transporter | -20.83 | |
| 27 | MGG_09019.6 | Secretory phospholipase A2 | -15.45 | |
| 28 | MGG_04386.6 | Urea amidolyase | -14.75 | |

| **MM – 10 most induced genes** | | | | |
| --- | --- | --- | --- | --- |
| **Rank** | **Gene Name** | **Annotation** | | **FC** |
| 1 | MGG_01041.6 | Cellulose signaling associated protein ENVOY | | 10.00 |
| 3 | MGG_07973.6 | Surface protein 1 | | 9.58 |
| 5 | MGG_08436.6 | Minor extracellular protease vpr | | 9.22 |
| 6 | MGG_10585.6 | Monooxygenase | | 8.11 |
| 7 | MGG_09352.6 | Minor extracellular protease vpr | | 7.90 |
| 8 | MGG_07146.6 | Glycosyl hydrolase | | 7.87 |
| 9 | MGG_09817.6 | Minor extracellular protease vpr | | 7.84 |
| 10 | MGG_06494.6 | D-arabinitol 2-dehydrogenase | | 7.51 |
| 15 | MGG_07791.6 | Surface protein 1 | | 6.67 |
| 17 | MGG_01281.6 | Glycerol kinase | | 6.64 |
| **MM – 10 most repressed genes** | | | | |
| **Rank** | **Gene Name** | **Annotation** | | **FC** |
| 1 | MGG_02201.6 | Endothiapepsin | -62.10 | |
| 4 | MGG_10715.6 | Zinc carboxypeptidase | -50.78 | |
| 6 | MGG_00460.6 | Rhamnolipids 3-oxoacyl-[acyl-carrier-protein] reductase | -33.57 | |
| 8 | MGG_05055.6 | Alcohol dehydrogenase | -30.38 | |
| 10 | MGG_07911.6 | Methyltransferase | -25.54 | |
| 17 | MGG_09019.6 | Secretory phospholipase A2 | -18.80 | |
| 23 | MGG_09990.6 | Minor extracellular protease vpr | -16.56 | |
| 27 | MGG_00018.6 | Integral membrane protein | -15.35 | |
| 31 | MGG_04386.6 | Urea amidolyase | -15.12 | |
| 33 | MGG_04194.6 | Acetyl esterase | -13.75 | |

| **MM-C – 10 most induced genes** | | | | |
| --- | --- | --- | --- | --- |
| **Rank** | **Gene Name** | **Annotation** | | **FC** |
| 1 | MGG_07868.6 | Endo-1,4-beta-xylanase | | 39.62 |
| 3 | MGG_10005.6 | Glycerol kinase | | 31.12 |
| 4 | MGG_00244.6 | 15-hydroxyprostaglandin dehydrogenase | | 27.04 |
| 5 | MGG_03880.6 | Alcohol dehydrogenase 1 | | 26.47 |
| 7 | MGG_09138.6 | Glutathione S-transferase II | | 25.05 |
| 8 | MGG_04345.6 | Cytochrome P450 17A1 | | 23.65 |
| 9 | MGG_07216.6 | Versicolorin reductase | | 22.67 |
| 11 | MGG_00994.6 | Mannosyl-oligosaccharide 1,2-alpha-mannosidase IC | | 22.62 |
| 13 | MGG_09139.6 | Laccase-1 | | 21.01 |
| 17 | MGG_05941.6 | Maltose permease MAL31 | | 20.15 |
| **MM-C – 10 most repressed genes** | | | | |
| **Rank** | **Gene Name** | **Annotation** | | **FC** |
| 6 | MGG_09019.6 | Secretory phospholipase A2 | -12.81 | |
| 7 | MGG_02235.6 | Oxidoreductase | -12.31 | |
| 8 | MGG_15047.6 | Major facilitator superfamily transporter | -12.17 | |
| 13 | MGG_10012.6 | Salicylate hydroxylase | -10.52 | |
| 14 | MGG_02201.6 | Endothiapepsin | -10.22 | |
| 19 | MGG_09893.6 | Molybdopterin synthase small subunit CnxG | -9.13 | |
| 22 | MGG_00419.6 | Major facilitator superfamily transporter | -8.77 | |
| 25 | MGG_01260.6 | Serine/threonine-protein kinase psk1 | -8.01 | |
| 28 | MGG_04401.6 | F-box protein | -7.70 | |
| 29 | MGG_12949.6 | GTP-binding protein 1 | -7.65 | |

| **MM-N – 10 most induced genes** | | | | |
| --- | --- | --- | --- | --- |
| **Rank** | **Gene Name** | **Annotation** | | **FC** |
| 3 | MGG_08670.6 | Choline transport protein | | 22.08 |
| 4 | MGG_05827.6 | Glutamyl-tRNA(Gln) amidotransferase subunit A | | 21.98 |
| 5 | MGG_05912.6 | N-acyl-L-amino acid amidohydrolase | | 20.74 |
| 6 | MGG_03533.6 | Formamidase | | 20.10 |
| 11 | MGG_07621.6 | Endoribonuclease L-PSP | | 17.84 |
| 12 | MGG_05828.6 | Chitin deacetylase 1 | | 17.57 |
| 16 | MGG_05871.6 | Integral membrane protein | | 15.87 |
| 18 | MGG_07627.6 | Homoserine acetyltransferase family protein | | 15.26 |
| 21 | MGG_13793.6 | Nitrate transporter | | 14.58 |
| 22 | MGG_08984.6 | Oligopeptide transporter 2 | | 14.23 |
| **MM-N – 10 most repressed genes** | | | | |
| **Rank** | **Gene Name** | **Annotation** | | **FC** |
| 3 | MGG_02201.6 | Endothiapepsin | -28.18 | |
| 10 | MGG_09019.6 | Secretory phospholipase A2 | -19.42 | |
| 14 | MGG_15047.6 | Major facilitator superfamily transporter | -18.62 | |
| 17 | MGG_10012.6 | Salicylate hydroxylase | -16.85 | |
| 19 | MGG_07911.6 | Methyltransferase | -16.31 | |
| 23 | MGG_10755.6 | Arylesterase/monooxygenase | -15.52 | |
| 28 | MGG_00419.6 | Major facilitator superfamily transporter | -14.02 | |
| 32 | MGG_02235.6 | Oxidoreductase | -13.04 | |
| 42 | MGG_02762.6 | ATP-dependent RNA helicase DED1 | -11.98 | |
| 44 | MGG_04401.6 | F-box protein | -11.74 | |
