# Supplementary material for: Transcriptome profiling of the rice blast fungus during invasive plant infection and in vitro stresses
Source: BMC Genomics. 2011 Jan 19;12:49. doi: 10.1186/1471-2164-12-49 (PMC3037901; doi:10.1186/1471-2164-12-49)
Supplement: Additional file 2 — Validation of the microarray experiments by quantitative RT-PCR. [file 1471-2164-12-49-S2.DOCX]

## Additional file 2 – qRT-PCR results for validation of *M. oryzae* microarrays. Microarray (M) and qRT-PCR (qPCR) fold-changes between the seven *M. oryzae* treatments in relation to *M. oryzae* grown in complete medium (reference sample) for 21 selected genes. Highlighted microarray and qRT-PCR fold-changes represent genes with opposite gene expression direction (more/less expressed than the reference).

|  |  | **R** | | **B** | | **TS** | | **PQ** | | **MM** | | **MM-C** | | **MM-N** | |
| --- | --- | --- | --- | --- | --- | --- | --- | --- | --- | --- | --- | --- | --- | --- | --- |
| **Gene** | **Description** | **M** | **qPCR** | **M** | **qPCR** | **M** | **qPCR** | **M** | **qPCR** | **M** | **qPCR** | **M** | **qPCR** | **M** | **qPCR** |
| MGG_09875.6 | CAS1 domain-containing protein 1 | 28.8 | 164.7 | 29.9 | 176.9 | 2.1 | 9.4 | 1.7 | 2.5 | 1.4 | 7.9 | 7.0 | 44.9 | 2.1 | 3.1 |
| MGG_09457.6 | Thymidylate kinase | -3.1 | -10.5 | -6.7 | -6.9 | -1.7 | 2.6 | 2.2 | 6.0 | -1.5 | 6.4 | 2.1 | 5.3 | 1.3 | 3.1 |
| MGG_07697.6 | Superoxide dismutase | 121.5 | 604.7 | 87.7 | 394.4 | 4.6 | 3.4 | 1.8 | 1.9 | 2.2 | -1.3 | 1.9 | 4.2 | -1.4 | -2.3 |
| MGG_08519.6 | Aflatoxin B1 aldehyde reductase | 147.2 | 370.5 | 91.7 | 362.9 | -1.6 | -1.4 | 3.1 | 2.7 | -1.1 | 23.2 | 11.7 | 32.4 | 1.9 | 1.4 |
| MGG_07868.6 | Endo-1,4-beta-xylanase | 86.8 | 128.3 | 387.5 | 7786.0 | 4.4 | -1.2 | 4.6 | 1.7 | 2.6 | 3.5 | 39.6 | 78.1 | 4.2 | 1.9 |
| MGG_10315.6 | Hydrophobin | 8.9 | 21.1 | 33.1 | 299.6 | -1.6 | 3.5 | -2.5 | 2.9 | -1.6 | 18.7 | 4.6 | 20.4 | 3.5 | 10.6 |
| MGG_02201.6 | Endothiapepsin | -1.7 | -4.0 | -18.3 | -99.5 | -80.5 | -1128.4 | -42.4 | -198.1 | -62.1 | -126.5 | -10.2 | -21.0 | -28.2 | -60.1 |
| MGG_03374.6 | Beta-1,6-galactanase | 13.8 | 12.0 | 20.8 | 87.2 | -1.9 | -3.6 | -1.5 | -1.3 | -1.6 | 1.0 | 3.3 | 4.3 | -1.3 | 1.6 |
| MGG_05719.6 | 30 kDa heat shock protein | -6.5 | -89.3 | -11.3 | -254.8 | -1.5 | -2.0 | 4.7 | 8.1 | -1.3 | 8.6 | 5.4 | 8.1 | 2.6 | 1.8 |
| MGG_09255.6 | Kinesin-II 85 kDa subunit | -3.8 | -3.4 | -6.5 | -6.5 | -9.4 | -3.1 | -6.6 | -1.6 | -7.2 | -2.0 | -6.4 | -2.3 | -4.8 | -2.2 |
| MGG_08918.6 | ATP-binding cassette sub-family G | -1.9 | -3.8 | -1.6 | -8.7 | 4.7 | 6.2 | 4.2 | 1.5 | 2.2 | 5.7 | 7.9 | 3.4 | 1.2 | 1.2 |
| MGG_09063.6 | Urea active transporter | -2.0 | -3.6 | -3.4 | -7.4 | -5.9 | -6.4 | -2.4 | -1.3 | -3.6 | 4.3 | -2.3 | -1.7 | 5.4 | 14.0 |
| MGG_06888.6 | Glutamine synthetase | -2.6 | -4.4 | -2.2 | -4.0 | -3.5 | -3.1 | -4.2 | -1.9 | -3.1 | -3.0 | -3.1 | -2.5 | -6.2 | -5.9 |
| MGG_07233.6 | Conserved hypothetical protein (potassium transporter) | -2.8 | -6.4 | -5.0 | -12.2 | -3.3 | -3.9 | -3.7 | -4.1 | -3.6 | -5.5 | -7.0 | -7.1 | -7.7 | -10.6 |
| MGG_03690.6 | Cholinephosphotransferase 1 | -3.4 | -5.9 | -3.3 | -5.3 | -5.4 | -5.4 | -6.7 | -5.2 | -5.6 | -3.1 | -3.7 | -2.9 | -3.3 | -3.5 |
| MGG_04404.6 | Pisatin demethylase | 39.8 | 670.9 | 52.6 | 40529.0 | 3.8 | 2817.1 | 6.9 | 3428.4 | 3.2 | 3176.7 | 11.8 | 10538.1 | 8.1 | 7332.0 |
| MGG_02393.6 | Cutinase | -1.2 | 4.3 | -2.2 | 1.9 | -2.8 | -1.5 | -7.6 | -1.2 | -2.6 | -1.1 | -1.6 | 1.3 | -3.4 | -1.7 |
| MGG_00312.6 | Glyoxylate reductase | -5.6 | 146.5 | -8.9 | 6073.6 | -4.0 | 23684.7 | -3.1 | 50535.2 | -4.0 | 91300.4 | -2.4 | 58996.2 | -2.1 | 52925.2 |
| MGG_07065.6 | G2/mitotic-specific cyclin-B | -1.2 | 16.6 | -1.3 | 82.5 | -2.3 | 28.8 | -2.8 | 23.4 | -2.2 | 20.9 | -3.4 | 16.1 | -1.4 | 42.1 |
| MGG_07912.6 | Erythrocyte band 7 integral membrane in | -2.8 | 1.8 | -4.7 | 1.9 | -4.6 | 4.3 | -4.1 | 8.0 | -4.3 | 8.1 | -2.8 | 5.7 | -4.9 | 2.5 |
| MGG_04401.6 | F-box protein | -3.3 | 6.0 | -6.3 | 10.9 | -10.7 | 10.4 | -11.7 | 5.0 | -10.5 | 4.9 | -7.7 | 4.6 | -11.7 | 3.8 |
| Validation average (73%) | | 76% | | 76% | | 67% | | 76% | | 52% | | 81% | | 81% | |
| Validation average without the last 4 genes (89.9%) | | 94% | | 94% | | 82% | | 94% | | 65% | | 100% | | 100% | |
