# Supplementary material for: Transcriptome profiling of the rice blast fungus during invasive plant infection and in vitro stresses
Source: BMC Genomics. 2011 Jan 19;12:49. doi: 10.1186/1471-2164-12-49 (PMC3037901; doi:10.1186/1471-2164-12-49)
Supplement: Additional file 4 — List of primers used in the validation of the microarray results by quantitative RT-PCR. [file 1471-2164-12-49-S4.DOCX]

**Additional file 4** - Sequence of primers used in the qRT-PCR reactions for validation.

| **Gene Name** | **Forward** | **Reverse** |
| --- | --- | --- |
| MGG_05587.6 | CTGCTGTCGCTCCGACATTAC | CCCGTCAAGTACGTCTCTCTCC |
| MGG_09875.6 | TTCCTCAGGTTTCTGCAGGTGGT | AGCAGTGCCATCCGAGTTGATCTT |
| MGG_09457.6 | TTGGGCGCAACGTCAAGGTTATGA | GAGCAAGTGAATGACATGGTCGTC |
| MGG_07697.6 | CGTTGACATGTGGGAGCATGC | GCCCTGGTGACGCGAACCTC |
| MGG_08519.6 | GCCGTCAGCGGTATGGGTC | CCCTCGTGTCCGGAGAGTG |
| MGG_07868.6 | GAGGGCGCCGCTCTCTTG | CAGCTTTGCGCTGCGCTC |
| MGG_10315.6 | CGTTCTCAGCGGCGAGTGC | GATAGGAGTGCACTGGATGTTGACC |
| MGG_02201.6 | GCGCCACGGTTATCACAGTCC | GTCTGCATCCGGCCGACG |
| MGG_03374.6 | CACAGCCAAGTCAACTCGTCTTCA | ACTTGCCGGTCGAAATGTGGTTGT |
| MGG_05719.6 | CTTTCTTCAGGAATTCCATCTACG | GACATCCTTTTCGCCGTTGTTG |
| MGG_09255.6 | CCGCTATACTCGGCTCCGACCAAA | CATCTTCTCCATCGCGGCCATTT |
| MGG_08918.6 | ACCGTCAAAGTGACCGATAGGGAT | AGCATGTTTAGGAGGGTCGTCTTG |
| MGG_09063.6 | GTCTTCGTGGACATGGGCGGCAA | GTCGCGAAGAGGATGATCTGCAC |
| MGG_06888.6 | TATGCGGGTGTTAAGATTTCGGG | GAGGAAGCGTGCTATTGAGAGGT |
| MGG_07233.6 | GCGGCTTCATGGTCGTGCCGATG | GTGCCCGGTACACGATGGACTTT |
| MGG_03690.6 | AATTGGCTTCATGTTCATTCTCGG | TGGTACATGAATAGACCGAAGGC |
| MGG_04404.6 | GGAAGCAGTTGGCTCTGATGG | CGGCATCCGCAACCCGTTC |
| MGG_02393.6 | GCGGTTTCCAAGGGGTTCTTTAG | CGAATACCCCGACAAGACAACCT |
| MGG_00312.6 | GCCGTTGCCCAGCAATCACAG | GTTCACCACGTTGACGAGCTC |
| MGG_07065.6 | AGAGGAGCAATGGGATGTCAGCAT | CGCATGGACCACTGTATCTCTGTT |
| MGG_07912.6 | GCCGATAACGTGACGCTCGAGCT | CGTCGTCTGGGCCAGCTGGGAGA |
| MGG_04401.6 | TTTGATGCGCATACTCTCAGGGTG | GCTGGATTTCCAAGAGCCAAACCA |
| MGG_01084.6 | CCTCGTCCATCTTCGACGC | GACACGACGGCTGTAGCC |
